# Antarctic Seabed Assemblages in an Ice-Shelf-Adjacent Polynya, Western Weddell Sea

**DOI:** 10.3390/biology11121705

**Published:** 2022-11-25

**Authors:** Bétina A. V. Frinault, Frazer D. W. Christie, Sarah E. Fawcett, Raquel F. Flynn, Katherine A. Hutchinson, Chloë M. J. Montes Strevens, Michelle L. Taylor, Lucy C. Woodall, David K. A. Barnes

**Affiliations:** 1 School of Geography and the Environment, Oxford University Centre for the Environment, University of Oxford, South Parks Road, Oxford OX1 3QY, UK; 2 Scott Polar Research Institute, University of Cambridge, Lensfield Road, Cambridge CB2 1ER, UK; 3 Department of Oceanography, University of Cape Town, Rondebosch, Cape Town 7700, South Africa; 4 Marine and Antarctic Research Centre for Innovation and Sustainability (MARIS), University of Cape Town, Rondebosch, Cape Town 7700, South Africa; 5 LOCEAN Laboratory, Sorbonne Université CNRS-IRD-MNHN, Jussieu, 75005 Paris, France; 6 School of Life Sciences, University of Essex, Wivenhoe Park, Colchester CO4 3SQ, UK; 7 Department of Biology, University of Oxford, Mansfield Road, Oxford OX1 3SZ, UK; 8 Nekton Foundation, Begbroke Science Park, Oxford OX5 1PF, UK; 9 British Antarctic Survey, High Cross, Madingley Road, Cambridge CB3 0ET, UK

**Keywords:** climate change, ice shelf, continental shelf, coastal polynya, benthic assemblages, benthic biodiversity, functional groups, Larsen C Ice Shelf, Antarctica, Weddell Sea

## Abstract

**Simple Summary:**

One-third of the Antarctic continental shelf is covered by ice shelves, floating extensions of the Antarctic Ice Sheet. Marine life beneath and bordering ice shelves is rarely investigated, yet likely to be highly impacted by climate change. As ice shelves retreat, marine environments transition into new open-water spaces, with potential for primary production and consequent food-fall to the seafloor. How Antarctic seabed assemblages (benthos) develop in such emerging spaces is influenced by neighboring and oceanographically-connected communities; thus, closing knowledge-gaps of benthic biodiversity near ice shelves underpins understanding of future ecosystem change. This study examined seafloor assemblages, and environmental differences, in a region that has experienced ice-shelf retreat, in a polynya adjacent to a marine margin at the forefront of climate change: the ice-shelf front. The study area, located in the Weddell Sea, is seldom accessible, and lies within a proposed international marine protected area. The study found a physically- and biologically-diverse seabed, complexity in potential environmental influences, and evidence of increasing megafaunal densities with increasing distance from an ice-shelf front. This research provides insights into seafloor habitats and inhabitants close to an evolving marine margin, and establishes ecological baselines from which biological responses to climate change can be evaluated to inform marine management.

**Abstract:**

Ice shelves cover ~1.6 million km^2^ of the Antarctic continental shelf and are sensitive indicators of climate change. With ice-shelf retreat, aphotic marine environments transform into new open-water spaces of photo-induced primary production and associated organic matter export to the benthos. Predicting how Antarctic seafloor assemblages may develop following ice-shelf loss requires knowledge of assemblages bordering the ice-shelf margins, which are relatively undocumented. This study investigated seafloor assemblages, by taxa and functional groups, in a coastal polynya adjacent to the Larsen C Ice Shelf front, western Weddell Sea. The study area is rarely accessed, at the frontline of climate change, and located within a CCAMLR-proposed international marine protected area. Four sites, ~1 to 16 km from the ice-shelf front, were explored for megabenthic assemblages, and potential environmental drivers of assemblage structures were assessed. Faunal density increased with distance from the ice shelf, with epifaunal deposit-feeders a surrogate for overall density trends. Faunal richness did not exhibit a significant pattern with distance from the ice shelf and was most variable at sites closest to the ice-shelf front. Faunal assemblages significantly differed in composition among sites, and those nearest to the ice shelf were the most dissimilar; however, ice-shelf proximity did not emerge as a significant driver of assemblage structure. Overall, the study found a biologically-diverse and complex seafloor environment close to an ice-shelf front and provides ecological baselines for monitoring benthic ecosystem responses to environmental change, supporting marine management.

## 1. Introduction

Previously long-constant Southern Ocean ecosystems are in transition, with climate change considered the greatest influencer [1,2]. Despite being geographically remote, and partially protected oceanographically by the Antarctic Circumpolar Current (ACC) for ~34 million years [3,4], the cold-water coastal seas of Antarctica are undergoing profound transformations, some of which are climate-linked [5,6]. These environmental transitions are perhaps most conspicuously evidenced by changes in the marine cryosphere (icescape), including areas of sea-ice decline, glacier recession, large iceberg calving and ice-shelf retreat and collapse, particularly in the Antarctic Peninsula and West Antarctic Ice Sheet regions [7,8,9,10,11,12,13]. Such ice losses are “blue-ing” the Southern Ocean, potentially opening up areas to new or enhanced primary production, and will likely have wide-ranging implications for Antarctica’s varied marine ecosystems, living resources, and ecosystem services over different spatial and temporal scales [6,14,15,16,17].

The majority (~95%) of Antarctica’s known marine biodiversity is seafloor-dwelling, i.e., benthic [18]. Benthos is a key component of the Southern Ocean ecosystem and food web [19], and is becoming increasingly recognized for its role in ocean health and carbon capture, storage and sequestration [20,21,22,23]. Antarctic seabed life (e.g., sponges, corals, brittle stars and feather stars) has evolved in partial isolation, afforded by the ACC (and its role as a barrier to meridional exchange), and relative constancy of physical conditions [1], punctuated by the seaward advance of the Antarctic Ice Sheet (AIS) during glaciations. Where such ice sheet extensions occur as grounded ice, much seafloor life is erased off the continental shelf. In contrast, the warmer interglacial periods are characterized by a comparatively much retreated AIS, and bring intense seasonality of light, sea-ice cover and food availability, and disturbance. Consequently, Southern Ocean seafloor biota is unlike any other, with very high levels of endemism—some taxa having undergone extensive evolutionary radiations (e.g., sea spiders and notothenioid fish) [1,24]. In comparison, other taxa are notably rare or absent (perhaps yet to be detected), such as durophagous (shell-crushing) predators (e.g., crabs and sharks; [25]). Furthermore, many benthic taxa possess specific adaptations and traits, exemplified by antifreeze glycoproteins [24], slow growth and development, and great longevity [1,26]. How such specialized biota and the communities they make up will respond to ongoing and anticipated changes in the Antarctic environment, including to a rapidly changing marine icescape, is, however, not yet clear [6,27,28,29].

Approximately one-third of Antarctica’s extensive (~4.6 million km^2^) and deep (mean depth > 450 m) continental shelf is overlain by a unique environment: ice shelves [26]. Ice shelves are the floating extensions of the AIS, often hundreds of meters thick and hundreds of km wide, and inhibit the flow of inland grounded ice into the ocean [13,30,31,32]. The marine cavities beneath ice shelves (i.e., sub-ice-shelf; between the grounding line and the ice-shelf front) experience aphotic conditions, some for thousands of years, and are arguably the least biologically-explored large-environment on Earth, in part due to challenges of access [33]. Benthic life beneath ice shelves has generally been posited as increasingly depauperate with increasing distance into the cavity [26,34]. This is hypothesized, in part, because sub-ice-shelf benthic communities, in particular the primary consumers, are considered as having some dependency on food supplies laterally advected under the ice shelf from open-water areas, although chemotrophic communities may also be present [35]. However, examples of sessile suspension feeders (e.g., sponges and bryozoans) have been observed as far as 260 km landward of an ice-shelf front, this being in addition to mobile scavengers and predators (e.g., [33,34,36,37,38]). These faunal observations suggest that such marine spaces are potentially more habitable than previously long-thought.

The remaining two-thirds of Antarctica’s continental shelf (~3 million km^2^) can be viewed as an “open-water” system, typically with regimes of seasonal sea-ice cover (extended winter sea-surface freezes and summer melts) and primary productivity (phytoplankton blooms) [1]; however, some open-water shelf areas, for example, in the Weddell Sea embayment, can be dominated by multi-year sea ice [39]. Although extensive sampling and knowledge gaps remain [40,41], far more is known about Antarctic open-water continental shelf communities. In this respect, rich and abundant benthic communities have been documented in some areas [42], as well as, more recently, expansive breeding colonies of icefish [43], while in other areas, communities shaped by intense disturbance from iceberg scouring or ploughing [44], and nearby glacier retreat [45] or rapid ice-shelf collapse [35].

Ice-related seafloor disturbance is a major driver of Antarctic shelf community patterns, and the severity and impact of such disturbances can differ over spatial scales, and temporally. For example, while iceberg scouring or ploughing of the seabed can be catastrophic to benthos at local scales, it can also, over time, promote higher regional diversity as mosaics of different faunal successional stages emerge across the seafloor [46,47,48,49,50]. A transfer from an ice-shelf-covered to open-water state, and vice versa, can also represent a disturbance [51], with energy dynamics considerably changing through the introduction of, for example, seasonal fluxes of light, and new occurrences of localized photosynthetic primary productivity [52,53]. Such changes can have implications for depositional and trophic regimes of benthic habitats, hence the structure of resident biotic communities [37,54]. Abrupt ice-shelf retreats, such as those associated with the 2002 disintegration of Larsen B Ice Shelf, eastern Antarctic Peninsula [55], can result in a forfeiture of some species living under the ice shelf, potentially accustomed to sub-ice-shelf environmental conditions. However, such environmental transitions can also create new opportunities for colonization of the seafloor and initiation of recruitment and community successional processes [56].

Following ice-related disturbance, (re)colonization of the Antarctic continental shelf can involve some predictable taxa and assemblage dynamics and, depth-depending, take from just several to hundreds of years for mature communities to (re)establish [46,48,57]. Faunal colonization, recruitment, and succession processes have been predominantly studied in the shallows near established research stations using settlement panels and SCUBA-based photographic surveys (e.g., [4,45,58,59]), and in deeper shelf waters using underwater cameras, towed collection apparatus and sediment cores (e.g., [60,61,62]). In such studies, the density and composition of proximate, or oceanographically-connected, communities are recognized as important to colonization and recovery patterns. However, temporal investigations of benthic-community dynamics following the retreat of an ice shelf above remain relatively rare, yet nevertheless include, for example, pivotal studies by Fillinger et al. [56] and Gutt et al. [54] at the Larsen Ice Shelf System, eastern Antarctic Peninsula—a region where pronounced ice-shelf loss has been observed over the satellite observation era [9,13,63].

Over the last 50 years, considerable ice-shelf loss has occurred in Antarctica, which is predicted to continue in a warming world. Indeed, a recent study by Gilbert and Kittel [64] has suggested that within this century, one-seventh to over one-third of Antarctica’s ice shelves are at risk of destabilization and potential collapse if the global mean atmospheric temperature reaches 1.5 to 4 °C above pre-industrial temperature, respectively. Furthermore, Gilbert and Kittel [64] also identified Larsen C Ice Shelf, once neighbored to the north by the now-collapsed Larsen A and B Ice Shelves, as one of the most at-risk and vulnerable ice shelves in Antarctica. With such future ice-shelf losses projected, it can be anticipated that there will be intensification in disruption to continental shelf environments, ecosystems, and associated biotic communities, particularly near to and underneath ice shelves, with potential implications for their structure, functioning and carbon sink capacity [20,21,23,44,65].

As a center of ice-shelf loss, and with the regional benthos well-studied by Antarctic standards (e.g., [35,54,56,66,67,68,69]), the eastern Antarctic Peninsula is a key region for studying how seafloor communities develop, over time, in response to a transition from once ice-shelf-covered to open-water coastal conditions. The current study explored four continental shelf sites adjacent to the Larsen C Ice Shelf front (a border between sub-ice-shelf and open-water shelf habitats), in an area falling within a proposed marine protected area (MPA): the Weddell Sea MPA (WS-MPA), under consideration by the Commission for the Conservation of Antarctic Marine Living Resources (CCAMLR) [70,71]. All study sites were within an occasionally-present coastal polynya and situated at various distances seaward from the ice-shelf edge, with two sites covered by ice shelf in recent-time. The study aimed to: (1) compare ice-shelf-adjacent benthic habitats and assemblages; (2) determine whether density and richness of benthos alter with distance from an ice-shelf front (and with substratum type), and subsequently discern potential functional group surrogates of the patterns observed (note, a surrogate is herein defined as a taxon that serves as a proxy for wider faunal patterns while not driving them); and (3) identify potential environmental drivers of assemblage structures discerned. A broader goal of the research was to increase understanding of the nature and variability of seabed habitats and life at the ice-shelf margins in areas at the cusp of environmental change. As such, the study provides an ecological baseline of benthic megafaunal structure from which change can be assessed and contributes to the wider understanding of the impact of a changing icescape on the benthos of the Antarctic continental shelf.

## 2. Materials and Methods

### 2.1. Study Area and Sites

The Weddell Sea lies within the Atlantic sector of the Southern Ocean. It is a coastal sea bordered by the Antarctic continent, including the Antarctic Peninsula, and the Scotia Arc, and is characterized by a broad and deep continental shelf (maximal depths exceeding 1 km), vast ice shelves, perennially-heavy sea-ice cover [26,39,72], and the presence of the strong cyclonic Weddell Gyre; the latter implicated in the northward transit of icebergs and sea ice along the eastern Antarctic Peninsula [73].

**Figure 1:**
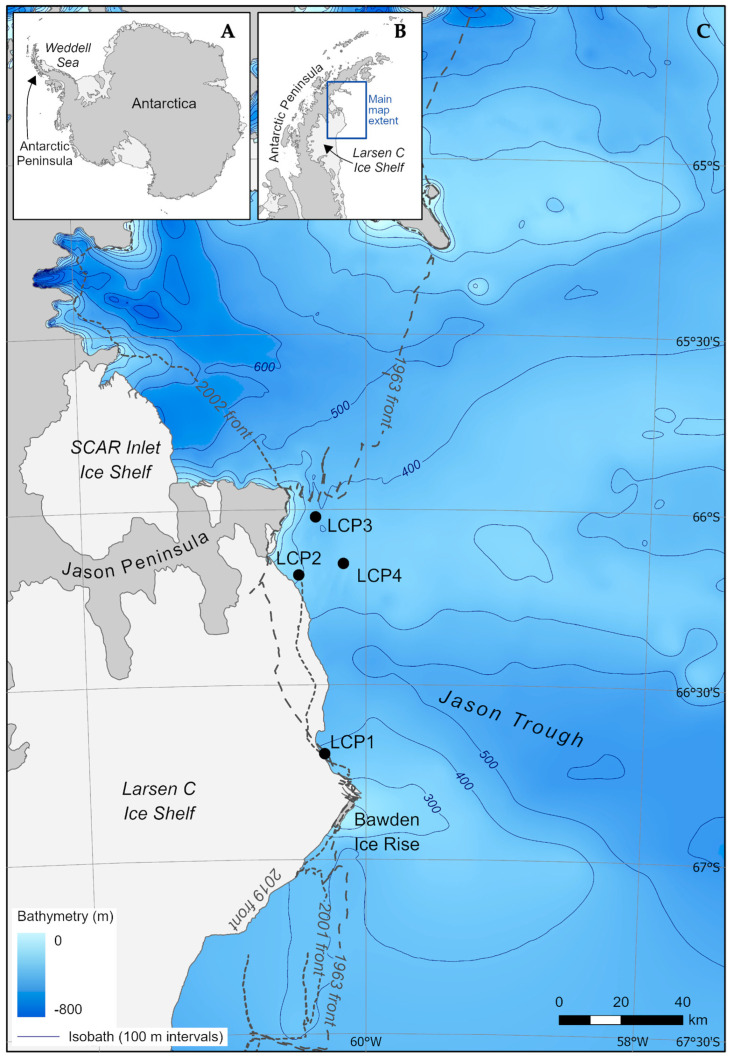
Study zone and sites. Inset (**A**) Antarctica indicating the Antarctic Peninsula and Weddell Sea; inset (**B**) Antarctic Peninsula indicating Larsen C Ice Shelf and the extent of the main map; and (**C**) main map of western Weddell Sea study area, showing the modern-day (2019) ice front and the locations of continental shelf study sites, LCP1-4 (black circles), investigated for benthic megafaunal assemblages. Light gray indicates ice shelves and darker gray indicates ice-covered land/grounded ice. Bathymetry is from the International Bathymetry Chart of the Southern Ocean v2 [79], with 100 m contours. The ice-shelf fronts are from Cook and Vaughan [9], Cook et al. [80] and Christie et al. [81].

The study area is situated on the inner continental shelf of the western Weddell Sea adjacent to the northeast sector of the Larsen C Ice Shelf (maximum width ~200 km and thickness of ice front ~200 m) [63,74], between Jason Peninsula and Bawden Ice Rise (Figure 1). It is within CCAMLR Statistical Subarea 48.5 and MPA Planning Domain 3, and proposed WS-MPA [70,71]. Associated fieldwork was carried out in January and February 2019 (austral summer 2018/19) aboard the South African polar supply and research vessel *SA Agulhas II* (IMO: 9577135), as part of the Weddell Sea Expedition 2019 data collection program [75]. At the time of sampling, study sites were located within the intermittent and wind-driven Larsen C Ice Shelf Polynya (LCP), averaging, when present, an open-water area of ~4700 km^2^ for 128 days per annum (pa), with phytoplankton blooms for 69 days pa [74]; the area is otherwise covered in first- to multi-year sea ice up to 5 m thick [39]. Benthic megafaunal assemblages were investigated at four sites, LCP1-4 (Table 1), situated at various distances from the nearest ice-shelf front, i.e., coastline (ranging from <1 to ~16 km, and labeled accordingly, with LCP1 being closest to the ice shelf and LCP4 farthest away). The sites were located on either side of the glacially-deepened Jason Trough [76,77,78]. LCP1 was the most southerly site, close to Bawden Ice Rise, and LCP2-4 were located north of Jason Trough, near Cape Framnes at the tip of Jason Peninsula. Sites were 15 to 75 km apart (Table S1), with LCP1 (the Bawden Ice Rise site) situated between ~57 to 75 km south of the Jason Peninsula sites, the latter all within ~19 km of each other. Sites were of depths ranging from ~300 to 400 m. At the time of sampling, a large tabular iceberg (A-68) was pivoting towards the study zone (see Figure 6A in Dowdeswell et al. [39]), in part shielding the area from the influx of sea ice and enabling vessel access.

### 2.2. Collation of Environmental Data Used in the Study

To discern how sites differed environmentally, and to help identify environmental variables potentially responsible for structuring megabenthic assemblages, various environmental datasets (remotely sensed, collected in situ and seafloor-imagery-derived) were used.

*Ice-shelf cover and ice-shelf front proximity.* The distance of each site from the nearest ice-shelf front, at the time of sampling, was calculated using Vector Analysis Tools in QGIS (see Table 1), and employed in the same way as distance from a coastline (or glacier front) is utilized (e.g., [42,82,83]). Historical areal extent and ice-shelf front positions of Larsen C (and its environs), available from 1963 onwards (with annual observations from 2009), were obtained from several published satellite-based datasets [9,80,81,84,85,86,87]. These datasets were used to estimate for each site for the 1963–2018 observation period, the number of years ice-shelf covered (or open water), the number of years since last covered by ice shelf, and when ice-shelf retreats occurred.

*Sea-ice concentration.* Mean monthly sea-ice concentration (SIC; %) data, spanning 1979 to 2019 and derived from Nimbus-7 SMMR and DMSP SSM/I-SSMIS passive microwave observations [88], were obtained from the US National Snow and Ice Data Center. These records have a grid cell resolution of 25 × 25-km^2^ and were processed in the same manner as described by Christie et al. [13]. Using this data, mean SIC was calculated for each site for the 1997–2018 period, complementing available primary production data.

*Primary productivity.* Mean daily net primary production (NPP; mg C m^−2^ day^−1^) data, spanning 1997 to 2018, were generated following the methods of Arrigo et al. [74,89]. From such data, both mean daily NPP and mean peak NPP were calculated for each site for the 1997–2018 period.

*Oceanography.* Oceanographical data were acquired by deployment of a CTD (conductivity-temperature-depth) system (Sea-Bird Scientific SBE 911plus), which included auxiliary sensors (e.g., oxygen and photosynthetically active radiation; PAR) and a rosette of 24 12-L Niskin bottles enabling collection of water samples (see Hutchinson et al. [90] and Flynn et al. [91]). The CTD was deployed from the sea surface to within 10 m of the seafloor at 19 stations in the study area. Measurements of temperature, salinity (from conductivity), and dissolved oxygen (to which a correction was applied, see [90]) were obtained, and from associated water samples, an array of nutrient concentrations determined (see [91]). For each site, data from the nearest CTD station were used, and associated measurements depth-matched wherever possible to the imagery; otherwise, the bottom-most measurements were utilized. CTD variables that varied negligibly between sites, including, for example, turbidity and PAR, were not included in further analyses, with those remaining given in Table S3.

*Substratum and phytodetritus.* Both substratum type (hardness) and level of phytodetritus cover were determined during seafloor imagery analysis (Section 2.5).

### 2.3. Seafloor Imagery Collection

At each site, three seafloor video-transects were conducted using a remotely operated vehicle (ROV) (EGI GP-50 Work Class; Eclipse Group Inc., Annapolis, MD, USA) (piloted by Eclipse Group Inc. and supported by Deep Ocean Search Ltd. and Ocean In-finity Inc.) equipped with a high-definition (HD) 1080i/29.97 fps color video camera (DSP&L HD Zoom SeaCam; DeepSea Inc., San Diego, CA, USA); the camera was forward-facing and angled obliquely towards the seabed. The ROV was fitted with two red parallel scaling lasers (DSP&L Micro SeaLaser; DeepSea Inc.), 10 cm apart, and six lights (DSP&L LED SeaLite; DeepSea Inc.). During dives, the three-dimensional position (i.e., latitude, longitude and depth) of the ROV was provided by a high-precision acoustic positioning system (Kongsberg HiPAP 502; Kongsberg, Norway). Videos were time-stamped, enabling the pairing of observations with positional data. Mean transect length was 847 m and depth within transects varied by <15 m.

### 2.4. Post-Processing of Collected Imagery

Raw transect videos (three per study site, 12 in total) were de-interlaced in HandBrake 1.3.3 (www.handbrake.fr, accessed on 15 June 2022), an open-source video transcoder. Frames were extracted from re-encoded videos every 20 s to avoid spatial overlap between successive frames. Frames not viable for analysis (e.g., too high above the seafloor, significantly obscured by sediment kick-up and/or laser points non-discernible) were discarded, the mean discard rate being ~25%. A 250 m sample was taken from each transect for analysis (and standardized to 100 m^2^ for richness calculations). Examples of seafloor imagery from the study are provided in Figure S1.

### 2.5. Frame Analysis and Assignments

As per Jones et al. [92], video frame appraisal methodologies were identical to those of still photographs. Frames were analyzed using the web-based imagery annotation tool BIIGLE 2.0 (www.biigle.de, accessed on 15 June 2022) [93]. Analysis was restricted to the lower-half of each frame (below the laser points), and frames were examined in random order. For each frame, all discernible epibenthic megafauna were annotated (hence counted) and identified to the highest taxonomic resolution possible or assigned a practicable morphotaxon/operational taxonomic unit (with taxonomic specialists and literature consulted, as appropriate, during the appraisal process). Substratum type was categorized as soft (i.e., mud or sand), assorted hard (varied coarse grain sizes from pebble to boulder (with soft sediment interstices)), boulder, or mixed sediment; the latter a ~50:50 mixture of soft and hard sediment. At sample-level, where frames became aggregated, substratum type assignment was based on the two most dominant substratum categories and then converted to a scale of “hardness” from 1–6 (1 being the softest substratum and 6 the hardest) (Table S2). The level of phytodetritus cover (indicated by a greenish layer on the seafloor) was recorded on a scale of 1–5 (1 being the lowest cover and 5 the highest). Laser points were marked and used to calculate the width of the field of view at the laser line, enabling computation of the seafloor area analyzed (mean of 2.2 m^2^ per frame). To provide further ecological context to the megabenthic diversity observed, and also to potentially identify ecological proxies to facilitate future monitoring, a functional group (one of 14; see Table 2), based on functional traits of feeding-strategy, mobility, and “skeletisation”, was ascribed to each morphotaxon; groups being adapted from Barnes and Sands [94] and Barnes et al. [95]. For each frame and sample, epibenthic megafauna and functional group counts were converted into densities, i.e., individuals per m^2^ (ind. m^−2^).

### 2.6. Statistical Analyses to Explore Biotic Differences and Potential Environmental Drivers

Statistical analysis comprised calculations of univariate indices, including the total number of morphotaxa (richness), as well as the application of multivariate statistical techniques in PRIMER v7 [98] with PERMANOVA+ add-on [99].

Prior to multivariate analysis, and adopting established procedures (see [98,100]; and references therein), environmental variables that displayed right-skewed distributions were log-transformed, and collinearity between variables was assessed using Draftsman plots. Of those variables that were highly correlated (or anticorrelated) (r ≥ 95% (or ≤−95%)), one variable was retained, acting as a proxy for the other(s) (Table S3). The remaining environmental variables were normalized to provide a common (dimensionless) scale for analysis. The megafaunal density data were fourth-root transformed to give more weight to rarer taxa and less weight to more dominant taxa [101].

The Bray–Curtis similarity coefficient [102] was used to evaluate compositional similarities of faunal assemblages within and between sites (based on the transformed faunal data), generating a resemblance matrix. Hierarchical (group-average) agglomerative clustering (HAC) analysis [103], based on Bray–Curtis similarities, was performed with a similarity profile (SIMPROF) test to visualize and assess the structure of the biological data, clustering the data into groups previously undefined and allowing for significant clusters to be identified [104]. The similarity percentages (SIMPER) routine [105] was employed to: (i) quantify the contribution of each morphotaxon to the average compositional similarity of samples within the SIMPROF-defined groups (in this case, groups = sites), i.e., identifying morphotaxa “typifying” groups; and (ii) quantify the contribution of each morphotaxon to the average compositional dissimilarity between groups, i.e., identifying morphotaxa “discriminating” among groups. Additionally, and also using Bray–Curtis similarities, a non-metric multidimensional scaling (nMDS) ordination [106] was used to visualize the compositional similarity of samples.

For the normalized environmental data, a resemblance matrix, based on Euclidean distance, was generated and used in a principal component analysis (PCA) [107]. The PCA was undertaken to visualize patterns in the environmental data across sites (and samples) and provide a preliminary indication of which variables may be influencing assemblage structures. In addition, the BEST (Bio-Env) routine, employing the Spearman’s rank correlation coefficient and including a global BEST test (999 random permutations) [104,108], was used to identify which subset of environmental variables best matched (or “explained”) the observed biotic patterns (i.e., assemblage structures), and to test the statistical significance of the match. Note, the procedure uses biological resemblance and normalized-environmental data matrices (and applies Euclidean distance to the latter during the process).

The statistical processes were also conducted for the functional group-based classification.

## 3. Results

### 3.1. Seafloor Habitat of the Study Area—Observations and Features

Across and within the study sites, the seafloor varied considerably in terms of rugosity (roughness) and dominant substratum type. Expanses of poorly-sorted coarse-grained (pebbles to boulders) substratum were particularly prominent at site LCP2, closest to Jason Peninsula. Such material appeared similar to that observed northward along the Larsen A Ice Shelf front [109]; indeed, the angularity and separateness of the boulders, in particular, suggested presence of supraglacially-derived ice-rafted debris [77].

Recent iceberg-scouring, such as that observed by Gutt and Piepenburg [49] and Gutt et al. [54] via ROV, was not evident in the imagery, although relatively wide and deep furrows were observed in the seabed at the two shallower sites, LCP2 and LCP4 (both <350 m deep).

Phytodetritus cover of the seafloor, indicative of potential “food banks” [110,111], was heaviest at the sites farthest offshore (i.e., LCP3 and LCP4), with lighter cover observed at the sites closest to the ice shelf, LCP1 and LCP2 (both within 3 km of Larsen C).

Circular/elliptic seabed depressions (~10 to 25 cm in diameter) were observed at each study site. These depressions appeared individually and in clusters, with more and larger clusters observed at the shallower sites, LCP2 and LCP4.

Very similar to the documented video-derived observations of Domack et al. [35] and Niemann et al. [112] of the continental shelf of the Larsen B embayment, potential chemotrophic habitats were observed. Such habitats presented as shallow indentations in the seabed (~0.75 to 2.5 m across) with soft sediment within (i.e., mud/sand), and were occasionally edged by bedrock. These habitats are likely inactive or low-activity cold seeps, evidenced by a faunistic legacy of *Calyptogena*-like shells and weedy worm-tubes (possibly Siboglinidae; see [113]), and the occurrence of potential bacterial mats within or around the features. Such chemotrophic habitats were observed at sites with the most contrasting ice-shelf-cover history, LCP1 and LCP4 (see Section 3.2), although further examples may have been camouflaged across the study area by sedimentation.

Across the study sites, biota, and associated functional groups, were, at times, highly variable in terms of abundance, diversity and composition—indicating a degree of faunal patchiness. Some areas could be depauperate, with little or no perceptible biota, or comparatively dominated by a particular taxon or functional group (e.g., corals and ophiuroids). In contrast, other areas could host multiple encrusting fauna (e.g., ascidians, sponges and pioneering bryozoans) on hard substratum (mainly larger rocks and boulders). Giant predatory and suspension-feeding asteroids, e.g., *Perknaster* sp. and brisingids, respectively, were observed in higher numbers at LCP2, the latter consistently on large boulders (potentially glacial dropstones). Instances of commensalism were evident throughout much of the study area, including, for example, crinoids and ophiuroids on top of corals or large sponges [114].

### 3.2. Environmental Differences Determined from Remotely-Sensed Data

*Ice-shelf cover and ice-shelf front proximity.* Observations of changing areal extent of Larsen C throughout the satellite era revealed varying degrees of ice-shelf cover over the study sites. Specifically, it was found that LCP1 and LCP2 had intermittently been covered by ice shelf in the observation period (1963–2018), consistent with the natural advance and calving of small icebergs along this particular stretch of the Larsen C front through time [9,13]. LCP1 and LCP2 were last ice-shelf-covered around 2009 and 2003, i.e., 10 and 16 years prior to sampling, respectively. It was observed that LCP1 had experienced (at least) two retreats, remained in close proximity (<1 to ~3 km) to the Larsen C ice-shelf front, and was ice-shelf-covered for (at least) two separate periods totaling 23 years (of the 56 years of observations). LCP2 also experienced two retreats and was covered for two distinct periods (totaling 7 years), and was proximate to Larsen C from the 1990s onwards. In contrast, LCP3 and LCP4 were free of ice-shelf cover throughout the observation period [13], and possibly for longer [115]. LCP3 was, in certain years, notably close to the Larsen B Ice Shelf margin (e.g., in 1968, 1986, and 1993), whereas LCP4 was comparatively (and consistently) distant from any ice-shelf fronts.

*Sea-ice concentration.* Mean monthly SIC (±1 SD), for the 1997–2018 period, was lowest at LCP1 (65.1% (±22.7)) and highest at LCP2-4 (69.6% (±25.7)). The low SIC values observed are indicative of the presence of the polynya.

*Primary productivity.* Mean daily NPP and mean peak NPP, for the 1997–2018 period, were highest at LCP4 and showed a significant increase with increasing distance from the ice-shelf front (regression-associated ANOVAs, R^2^ = 0.93 and 0.94, F = 129.37 and 152.16, respectively, with both *p*-values < 0.001). For LCP1-4, the mean daily NPP values (±1 SD) were 123.5 (±202.8), 165.9 (±226.4), 199.4 (±245.2), and 243.8 (±309.3) mg C m^−2^ d^−1^, respectively, and mean peak NPP values (±1 SD) 264.5 (±329.7), 372.6 (±207.7), 435.4 (± 309.5), and 791.7 (±552.9) mg C m^−2^ d^−1^, respectively.

### 3.3. Megafaunal Richness: Accumulation with Area, and Pattern with Ice-Shelf Proximity and Substratum Hardness

Overall, a total of 98 morphotaxa from 23 classes of 10 phyla were observed across the study zone.

**Figure 2:**
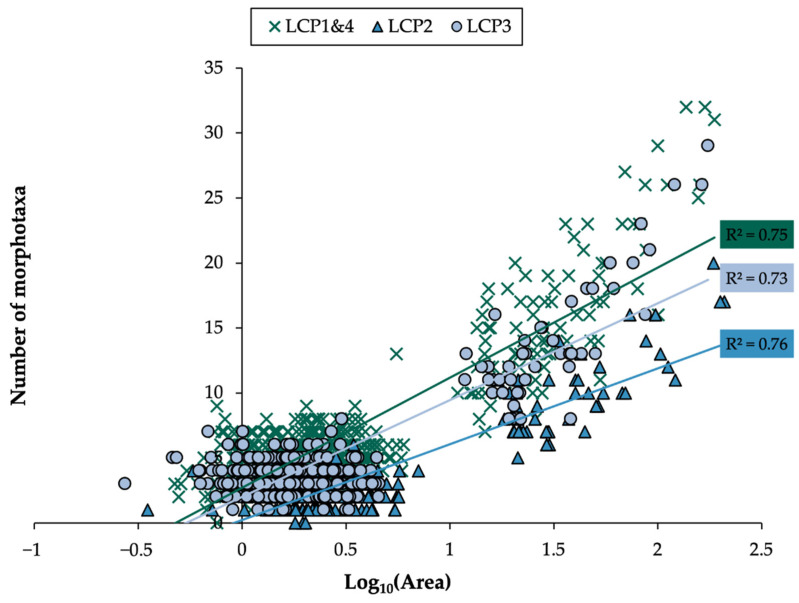
Ice-shelf-adjacent sites accumulate faunal richness differently. Morphotaxa richness with area surveyed for sites LCP1-4 (note, slopes for LCP1 and LCP4 were not significantly different from each other, *p* > 0.2, therefore, datasets pooled). Significantly different slopes are shown (*p*-values for differences between slopes all <0.01). For regression lines, F-values = 1546.39, 767.44, and 930.35, for LCP1 and LCP4 combined, LCP2, and LCP3, respectively, with *p*-values all <0.001.

The relationship between richness and area surveyed was examined at each site and compared to determine if megafaunal richness accumulated differently across sites. As expected, faunal richness increased from the area of a single frame to a sample (mean 165.7 m^2^), the latter peaking at 32 morphotaxa (Figure 2). The lowest intercept and most gradual slope (indicative of richness accumulation) were at LCP2, equating to half the level of richness observed at LCP1 and LCP4 (the sites closest to and farthest from the ice-shelf edge, respectively). LCP1 and LCP4 were not significantly different in terms of richness accumulation (*p* > 0.2), while all other pairwise comparisons indicated slopes to be significantly different.

Larger areas increasingly accumulated the less abundant and rarer morphotaxa (e.g., particular sponges, asteroids, anthozoans, ascidians, and brachiopods), including “singletons” (morphotaxa that only appeared once), and this mainly drove the increased richness observed at LCP1 and LCP4, with different taxa contributing to the morphotaxa count of each site.

When comparing standardized 100 m^2^ samples (three per site), the lowest richness was observed in an LCP2 sample and the highest in an LCP4 sample, with 11 and 31 morphotaxa recorded, respectively. An LCP1 sample exhibited the second-highest richness at 27 different morphotaxa. The richness of LCP4 samples was varied, with the value of one sample being almost double of another (16 and 31), whereas the richness of LCP3 samples was noticeably more uniform (23, 24 and 25). Marked variability in richness was found at the sites closest to the ice-shelf front, with richness more than doubling from the poorest to richest sample (11 and 27).

In terms of a relationship with proximity to the ice shelf, richness did not significantly alter with increasing distance from the ice-shelf front (regression-associated ANOVA, F = 1.0, *p* = 0.33). However, when LCP1 samples were excluded from the exploration, as they were located within a different water mass (see [90]), the relationship became more robust although remained non-significant (R^2^ value = 0.4, and regression-associated ANOVA, F = 4.6, *p* = 0.06).

Overall, a weak (inverse) relationship was observed between richness and “hardness” of substratum (Figure S2, F = 6.59, *p* = 0.03), although richness did halve from predominantly soft-sedimented (with incidences of mixed) substratum through to assorted hard sediment-boulder fields.

### 3.4. Megafaunal Density and Pattern with Ice-Shelf Proximity and Substratum Hardness

**Figure 3:**
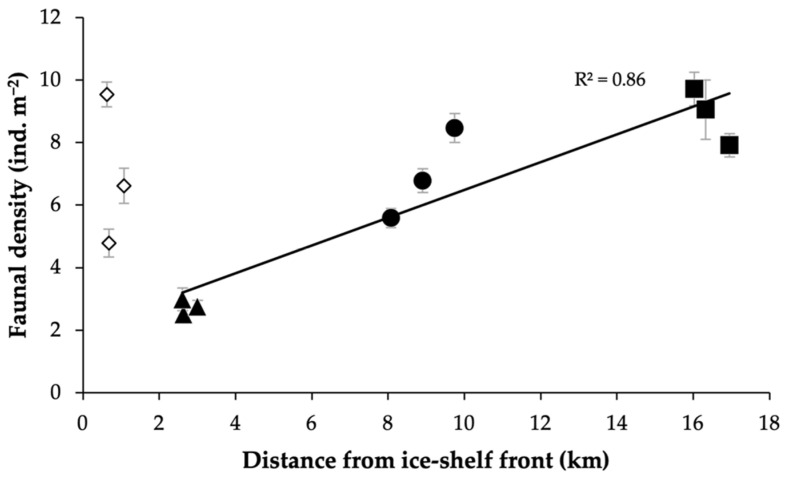
Megafaunal density generally increases with distance from the ice shelf. Mean benthic megafaunal density (ind. m^−2^) (±SE) of each sample (three per site) with distance from the ice-shelf front (km). Mean density (±SE) calculated for each sample using associated frames (minimum of 44 frames per data point). Site LCP1 = diamonds, LCP2 = triangles, LCP3 = circles, and LCP4 = squares. Symbols filled in white excluded from regression statistics (hence trend-line generation) (owing to water mass differences, see [90]).

Across samples, mean benthic megafaunal density varied by nearly an order of magnitude, from 2.5 to 9.7 ind. m^−2^ (see y-axis of Figure 3). The lowest densities were typically found at LCP2 (all three samples observed to be faunally depauperate) and the highest densities generally at LCP4; although, at LCP1, closest to the ice-shelf edge, faunal densities could also be high, although more variable. When considered together, the two sites nearest to the ice shelf were more variable in terms of faunal density than the two sites farthest away.

With increasing distance from the ice-shelf front, it was observed that density generally increased (regression-associated ANOVA, R^2^ = 0.35, F = 5.39, *p* = 0.04). To control for water mass differences, this trend was also explored without LCP1, where it became more significant (Figure 3, regression-associated ANOVA, F = 43.3, *p* < 0.01).

The general relationship of increased faunal density from the ice-shelf front to farther offshore was principally caused by increases in densities of bottlebrush corals (e.g., Primnoidae, including *Thouarella* spp.), ophiuroids (e.g., *Ophionotus*) and encrusting lithophilic fauna (e.g., pioneering bryozoans, ascidians and sponges). Bottlebrush corals and encrusting fauna mainly drove the high variability in density and occasionally high values at LCP1.

**Figure 4:**
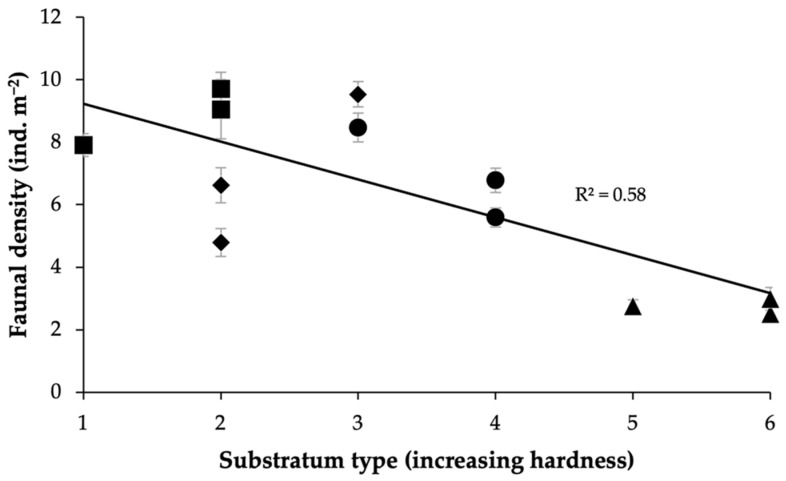
Megafaunal density generally decreases with increasing hardness of the substratum. Mean faunal densities (ind. m^−2^) (± SE) of samples with substratum type (scale of increasing hardness from left to right), where 1 = soft-sediment: mixed; 2 = mixed: soft-sediment; 3 = mixed: boulder; 4 = assorted hard: mixed; 5 = assorted hard: boulder/mixed; and 6 = assorted hard: boulder. Site LCP1 = diamonds, LCP2 = triangles, LCP3 = circles, and LCP4 = squares.

The nature of the substratum also appeared to influence faunal densities, with faunal density significantly decreasing with increasing substrate hardness (Figure 4, regression-associated ANOVA, F = 13.7, *p* < 0.01). The lowest density values were observed in the samples categorized as having the “hardest” substratum type. This trend was principally driven by decreased densities across the majority of faunal groups at LCP2 (closest to Jason Peninsula), but particularly in encrusting suspension feeders, and the apparent absence of certain taxa, including bottlebrush corals (see Section 3.6).

### 3.5. Functional Group Richness and Density Patterns with Ice-Shelf Proximity and Substratum Hardness, and Potential Surrogacy of Wider Faunal Patterns

Of the 14 functional groups (Table 2), 12 were represented by the fauna discerned in the imagery. Whether such functional groups reflected taxonomic patterns in richness and overall density was assessed.

With respect to richness, functional group richness, like morphotaxa richness, did not significantly change with increasing distance from the ice-shelf front, including when removing groups represented by singletons (both *p*-values > 0.3). However, in contrast, functional group richness did not exhibit a relationship with substratum hardness, including when removing singleton groups (both *p*-values > 0.7).

**Figure 5:**
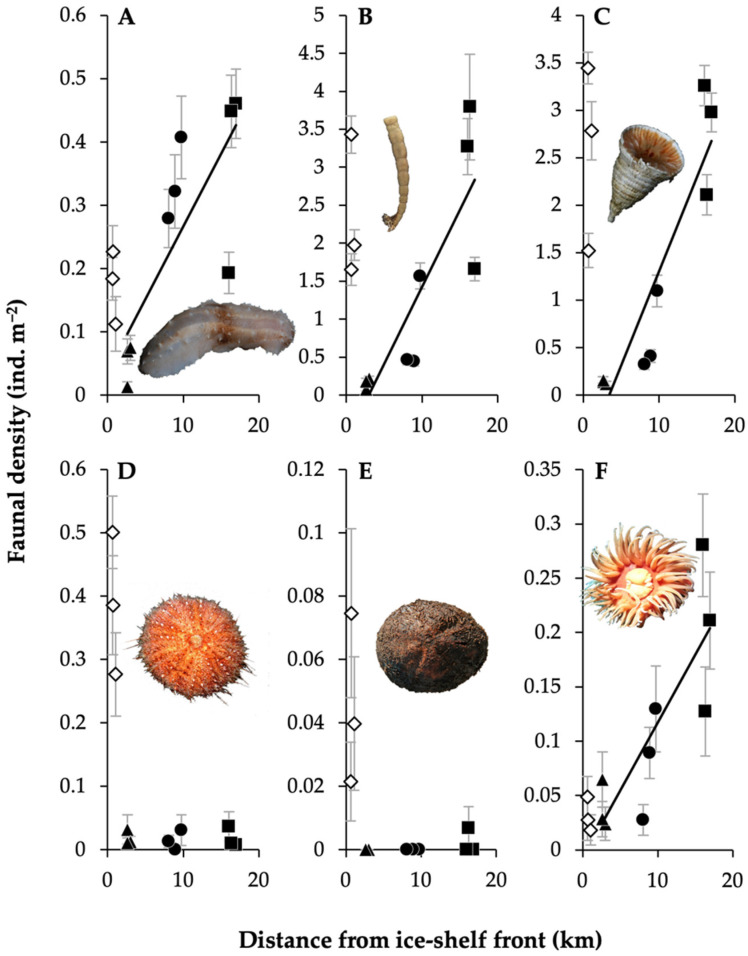
Functional group densities can alter differently relative to ice-shelf proximity. Mean functional group densities (ind. m^−2^) (±SE) of samples with distance from the ice-shelf front (km), with an example taxon for each group (images provided by D.K.A.B.): (**A**) deposit-feeding crawlers; (**B**) pioneer sessile suspension feeders; (**C**) hard sessile predator/scavengers; (**D**) grazers; (**E**) hard deposit-feeders; and (**F**) soft sessile predator/scavengers. Site LCP1 = diamonds, LCP2 = triangles, LCP3 = circles, and LCP4 = squares; white-filled symbols indicate (due to water mass disparities, see [90]) data not included in trend-line generation. R^2^ values for (**A**–**C**,**F**) = 0.63, 0.85, 0.74 and 0.70, respectively, and regression statistics indicated all associated *p*-values ≤ 0.01.

As with taxonomic groups, the density of some functional groups altered with distance from the ice shelf. Deposit-feeding crawlers (all holothurians), pioneer sessile suspension feeders (primarily encrusting lithophiles) and hard sessile predator/scavengers (principally bottlebrush corals) all showed similar patterns to the overall faunal trend (Figure 5A–C); this including high densities at LCP1 (cf. Figure 3). In contrast, grazers and hard deposit-feeders (both groups predominantly echinoids) showed high densities at LCP1 and no trend with increased distance away from the ice shelf (Figure 5D,E). Some fauna, such as soft sessile predator/scavengers (primarily anemones), increased in density with distance from the ice shelf (Figure 5F) and did not display high densities at LCP1 (hence, differed from the overall faunal pattern).

With regard to substratum type, deposit-feeding crawlers, hard sessile predator/scavengers, and pioneer sessile suspension feeders, like the overall fauna pattern, decreased in density with increased substratum hardness (regression-associated ANOVAs, F = 7.4, 19.88 and 11.64, *p* = 0.02, 0.001 and 0.007, respectively). Whereas other functional groups (and component taxa), e.g., climax suspension feeders, flexible strategists (all ophiuroids), sedentary suspension feeders (all crinoids), and mobile hard predator/scavengers (principally asteroids and notothenioid fish) did not show clear trends with substratum hardness (*p*-values > 0.1).

**Figure 6:**
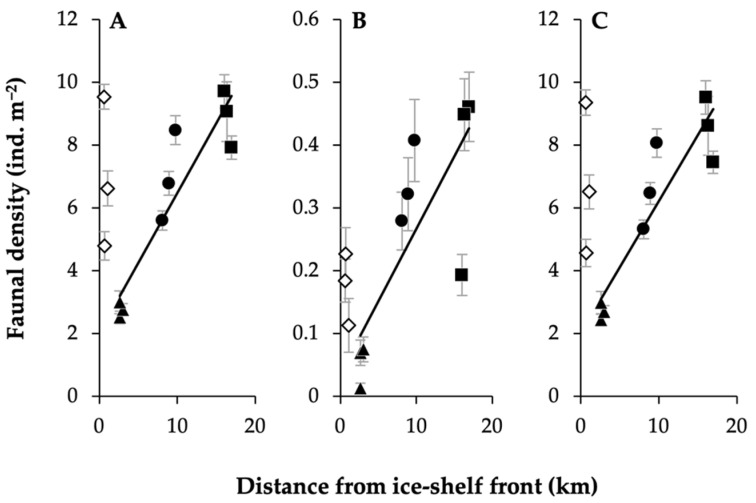
Deposit-feeding crawlers as an effective surrogate of the overall faunal density trend observed with distance from the ice shelf. Mean densities (ind. m^−2^) (±SE) with distance from the ice-shelf front (km) of: (**A**) overall fauna; (**B**) deposit-feeding crawlers; and (**C**) overall fauna excluding deposit-feeding crawlers. Site LCP1 = diamonds, LCP2 = triangles, LCP3 = circles, and LCP4 = squares; white-filled symbols indicate data not included in trend-line generation (because of water mass differences, see [90]). R^2^ values for (**A**–**C**) = 0.86, 0.63 and 0.85, respectively. Regression statistics for C presented a *p*-value of ≤0.001.

On exploring whether any functional groups could be considered a proxy (surrogate) for the wider faunal patterns observed, e.g., in density, deposit-feeding crawlers appeared to be the strongest surrogacy candidate (Figure 6; and see Figure S3 for pioneer sessile suspension feeders and hard sessile predator/scavengers). The functional group individually reflected the density pattern, and when removed from the broader dataset, the pattern remained, supporting that deposit-feeding crawlers did not drive the trend. Furthermore, and as above-mentioned, the density-trend of deposit-feeding crawlers with substratum type also reflected that of the overall fauna.

### 3.6. Megafaunal Assemblage Composition

The nMDS ordination of megafaunal assemblages of samples showed a robust two-dimensional representation of non-metric multidimensional space (stress 0.05, Figure S4). This analysis revealed that the sites closest to the ice shelf were not compositionally homogeneous, in fact LCP1 and LCP2 had the most dissimilar assemblages (61.2% dissimilarity) of any two sites. The highest Bray–Curtis similarities were observed from intra-site comparisons (highest = 77.6% at LCP4) and the lowest from inter-site comparisons (lowest = 35.6% between LCP1 and LCP2). The key morphotaxa found to be driving similarity between samples within sites were ophiuroids (particularly for LCP2 and LCP3), encrusting lithophiles and bottlebrush corals (Table S4). Key morphotaxa driving dissimilarity between sites included bottlebrush corals, particularly when comparing LCP2 samples with others (although not key for LCP1 and LCP4 dissimilarity), *Sterechinus antarcticus* (as well as other echinoid species), principally when comparing LCP1 with offshore sites, and, in some site comparisons, encrusting lithophilic organisms and *Pyura* sp. (potentially *Pyura bouvetensis*) (see Table S5).

The differences between LCP2 samples and those of other sites appeared to be driven by absences and much lower densities of various taxa, although LCP2 was the only site in which the holothurian *Peniagone vignoni* was observed. Notably, LCP1 had higher densities of echinoids (of at least three different morphospecies). At LCP4, an athecate hydroid species occurred in higher densities than at other sites. Compared to LCP3, LCP4 had more bottlebrush corals, the aforementioned hydroid species, and the *Pyura* sp. In general, LCP3 and LCP4 had higher densities of holothurians (of differing species), particularly those (visually) identified as *Pseudostichopus mollis*.

**Figure 7:**
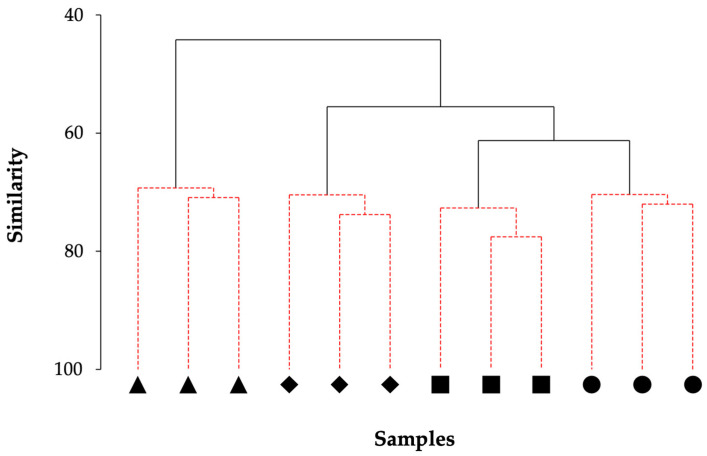
Clustering of samples corresponds with respective study site. Dendrogram resulting from hierarchical cluster analysis of samples from Larsen C study sites (based on Bray–Curtis similarities and using group-average linking). Black continuous lines indicate significant clusters, identified by a SIMPROF test, and red dashed lines indicate non-significant clusters. Site LCP1 = diamonds, LCP2 = triangles, LCP3 = circles, and LCP4 = squares.

HAC analysis with a SIMPROF test identified four distinct groupings among samples, corresponding to the four individual sites (Figure 7). Further examination of the HAC dendrogram showed that LCP2 samples were compositionally more distinct from those of the other sites; this was followed by LCP1, and then LCP3 and LCP4. The results of this analysis were reflected in the nMDS findings showing that the sites closest to the ice-shelf front were the most compositionally dissimilar.

When HAC (plus SIMPROF) analysis was performed for functional groups, results were similar to that of the morphotaxa in that the samples were again clustered by their respective study site (Figure S5).

### 3.7. Environmental Differences between Study Sites

**Figure 8:**
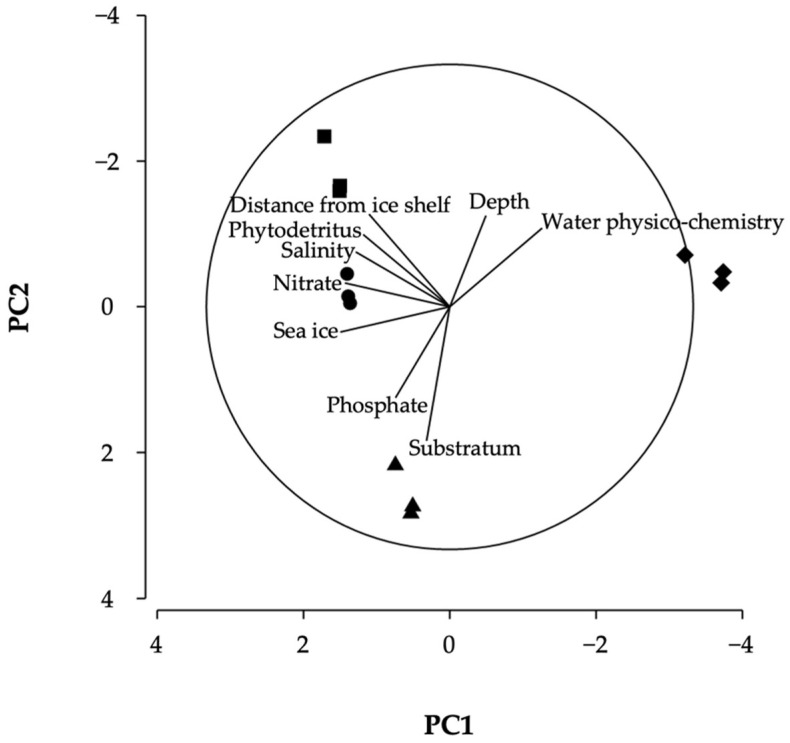
Differences in the environmental setting of sites (and samples). PCA ordination showing spatial variation in environmental variables among the 12 samples. Site LCP1 = diamonds, LCP2 = triangles, LCP3 = circles, and LCP4 = squares. PC = Principal Component.

A range of environmental variables (15 in total and 9 following correlation analysis, see Table S3) was examined to discern how sites differed in terms of environmental context. The PCA and associated ordination (Figure 8) showed that the first two principal components accounted for >85% of the environmental variability (52.9% and 32.4%, respectively). The first principal component explained environmental differences among samples due to sea-ice cover, nitrate, salinity, and water physico-chemistry (represented by temperature and correlation with oxygen concentration) (Eigenvectors = 0.45, 0.43, 0.39, and −0.38, respectively). The second principal component further showed the factors of substratum type, distance from the ice-shelf front, depth, and phosphate (Eigenvectors = −0.55, 0.38, 0.38 and −0.38, respectively).

Overall, the PCA indicated the importance of measuring a range of environmental variables when studying multiple sites, as water physio-chemistry appeared the most influential in differentiating LCP1 from the other sites (hence, separation in Figure 3), substratum at LCP2, and distance from the ice shelf and phytodetritus cover at LCP3 and LCP4.

### 3.8. Environmental Influences on Megafaunal Assemblage Composition Differences

Having established environmental differences among sites, BEST analysis was performed to determine which variables correlated with, and hence could be potentially influencing, megabenthic assemblage compositional differences in space. The results showed that specific combinations of environmental variables were more likely to be underpinning faunal differences.

The BEST analysis indicated that a subset of three environmental variables most highly correlated with (or “best explained”) the variation in faunal assemblage structure (Rho correlation = 0.93, *p* < 0.01). This subset included mean depth, water physico-chemistry and substratum hardness. The single variable showing the highest Rho correlation (0.77) was substratum type, considered a local-scale variable.

When comparing BEST analysis results of all faunal grouping methods, i.e., morphotaxa, class and functional groups, the most highly correlated subsets of potential explanatory variables each included water physico-chemistry and substratum hardness (Rho correlations for the latter two faunal groupings = 0.87 and 0.93, respectively, with associated *p*-values < 0.01). As such, there appears to be some consistency in which environmental variables emerged as potential key drivers of the biotic variation observed, irrespective of whether the benthic biodiversity was classified by taxonomic levels or functional groups.

## 4. Discussion

The Southern Ocean can be characterized by relative constancy [1] and also sharp marine environmental gradients, the latter exemplified by conditions either side of the Polar Front (the strongest jet of the ACC) [116], the continental shelf break (the terminus of grounded ice in glaciations) [72,117,118], the marginal sea-ice zone and the ice-shelf front [119]. With ocean and atmospheric warming, Antarctic icescapes and associated marine ecosystems are expected to change, with implications for biota [2,6]. While there have been various studies of biological response to Antarctic glacier retreat (e.g., [45,120,121,122]), few have covered life underneath ice shelves (see [33,34,38]) and even fewer on what shapes biodiversity, and how it develops, in areas that have recently experienced ice-shelf loss [54,56,68]. The current study investigated megabenthic biodiversity (richness, density and composition) and potential environmental drivers at a key Antarctic marine frontier that is arguably one of the most sensitive to climate change, the ice-shelf edge. Furthermore, the respective study area sits entirely within the constraints of an important and characteristic Antarctic marine feature, a coastal polynya [74,123]. The seafloor biology where these two systems co-occur is little known [124], and yet, potentially representative of how seabed communities may develop in new areas of open water as Antarctic ice shelves retreat in a warming world.

The present study found a trend in the overall density of megafauna with increasing distance from the ice-shelf front; however, perhaps surprisingly, not in richness (biodiversity). When individual taxa and functional groups were explored, some groups were found to reflect the overall density trend (i.e., could potentially be used as proxies), some contrasted, and some showed no clear pattern. Substratum type appeared to strongly influence megafaunal density, although it did not appear to exert the same impact on richness. Distance from the ice-shelf edge, which, in the confines of the study, correlated with the remotely-sensed primary-production-related variables, did not emerge as a significant environmental driver of assemblage composition. Instead, different environmental variables appeared to be important locally at each study site. Compared to offshore sites, assemblages closest to the ice-shelf front could be expected to be more similar to each other (having had less time in terms of environmental exposure to diverge), yet they were actually the most dissimilar. Overall, results suggested that a study zone, where a polynya abuts the ice shelf, can be a complex coastal environment, both physically and biologically.

**Figure 9:**
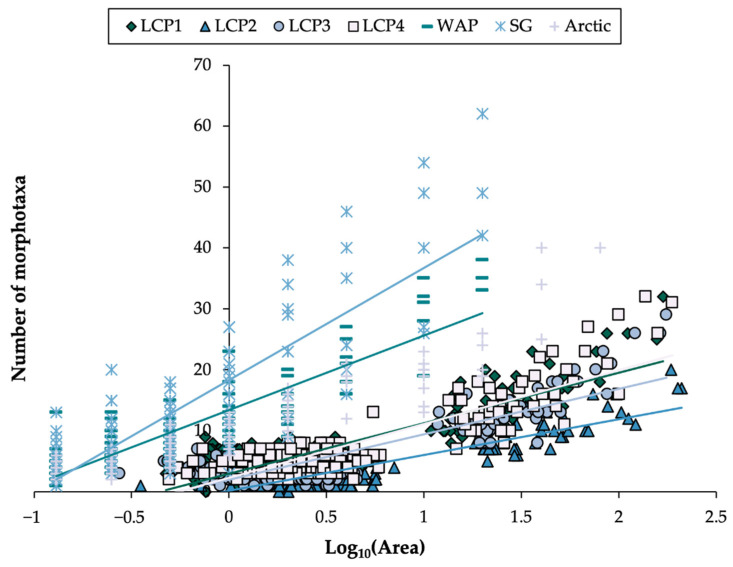
Geographical regions accumulate faunal richness differently. Morphotaxa richness with area examined for Larsen C Polynya study sites, LCP1-4, compared to photographic studies from Ryder Bay (western Antarctic Peninsula, WAP), around the island of South Georgia Island (SG) and the Barents Sea (labeled here as “Arctic”) (sources of external datasets used are provided in the text).

Large-scale alterations in faunal richness have been widely observed across geological time [125] and space, e.g., latitude [126,127], with pronounced hemispherical asymmetry [1,128] due to the Antarctic Ocean being anomalously highly biodiverse [18,26]. Various reasons for changes to faunal richness across scales have been presented, including energy availability [129] and species turnover [130]. Given that the number of species encountered generally increases with the size of area surveyed (e.g., Figure 2), it is important to evaluate richness levels within comparable area sizes (i.e., standardized samples), for example, when comparing findings, such as the current study’s, with those of other regions. In the case of the present study, investigating continental shelf assemblages proximate to Larsen C, morphotaxa richness increased (at each site) with the size of area examined, however, less rapidly than, for example, at Ryder Bay (western Antarctic Peninsula) [21], around the island of South Georgia [94], and in the Barents Sea (Arctic) [131] (Figure 9, using associated datasets).

Potentially having experienced greater historic disturbance (e.g., from iceberg scouring), Antarctica’s coastal open-water shelf areas can harbor a great array of habitat types and niches, and hence resident biota, including those associated with different successional stages. As such, it was envisaged that a positive richness gradient would be observed with increasing distance from the ice-shelf front (i.e., farther offshore). However, within the study confines, no significant increase (or change) in faunal richness was discerned (including when examining Jason Peninsula sites exclusively). Furthermore, richness values were particularly variable at the sites closest to the ice-shelf front. Similarly, Grange and Smith [132] found no increase in richness with distance from western Antarctic Peninsula glacier termini, whereas Kim et al. [83] found the greatest richness at a location farthest from a glacier front in Marian Cove, King George Island. The results of the current study could be due to several reasons, including: (1) the nature of the seabed environment close to the ice-shelf front being similarly (or more) variable to that of open shelf farther away (e.g., reflected in the different prevailing substratum types); and, alternatively or additionally, (2) the principal environmental influences driving biodiversity patterns are site-specific, i.e., differ according to the site (Figure 8; and as found by Post et al. [133] with regard to rugosity), and are more influential than ice-shelf-edge proximity (and respective correlates, i.e., mean daily and mean peak NPP). However, examining biodiversity differences using richness alone can obscure underlying variances and changes [134] and is better evaluated in coordination with other measures such as density and faunal composition.

Biota responses to major environmental gradients can be reflected in faunal density in both space and time. In the polar regions, this can be exemplified by (re)colonization of seafloor on ecological timescales in response to ice scour, glacier retreat and ice-shelf collapse [44,54,56,132,135], and by (re)colonization of the continental shelf on evolutionary timescales following the recession of the AIS and associated grounded ice (e.g., following the Last Glacial Maximum ~20,000 years ago) [136,137]. Significant changes in the density of benthos can occur from the abyssal plain through continental slope to continental shelf [118], i.e., spatially, and over time, for example, in association with food-fall [111,138]. However, some studies have shown that faunal abundance does not always directly reflect observed phytodetritus fall and cover of the seafloor [139,140]. The present study found that faunal density generally increased with increasing distance offshore. This result is in some contrast to the findings of Grange and Smith [132], which showed lower densities on the open-water continental shelf compared to study locations at glacial termini, albeit on a larger spatial scale. However, in the current study, the Bawden Ice Rise site (LCP1), closest to the ice shelf and with the greatest ice-shelf cover over the observation period, had some of the highest faunal densities observed in the study area. These high densities may have been driven by the site experiencing different water mass conditions (see Figure 3 and Figure 8, and [90]); in particular, water temperatures were higher, possibly influencing the occurrence and growth of taxa (e.g., see [27]) and/or parts of the site being serviced by bottom currents rich in organic matter.

Additionally, the seafloor (hence benthos) at LCP1 may have been more protected from ice-mediated disturbance than the Jason Peninsula sites due to being more consistently ice-shelf-covered, and for a more extended period, at least within the satellite era. Furthermore, when open water, LCP1 may have been afforded further protection from disturbance by Bawden Ice Rise and associated shallow seafloor, known to constrain iceberg-flow into and within the area [141]. Nonetheless, it is notable that farther north on the western Weddell Sea continental shelf, it has been observed that in just a few years of areas becoming ice-shelf-free, density and biomass of benthos can increase considerably, i.e., in response to new exposure to open-water conditions and associated food regime changes [54,56]. 

The site with the lowest faunal densities was LCP2, closest to Jason Peninsula. Such density values may be explained in reference to the aerial/satellite imagery that showed the site had often been proximate to the ice-shelf edge and had undergone ice-shelf advance and retreat (at least twice) in recent-time. Additionally, the site is at a water depth within reach of modern iceberg keels in an area of iceberg transit (conditions also particularly pertinent to LCP4 and potentially explaining the faunal patchiness observed there). Hence, the respective site may have been especially subject to disturbance such as scouring and the deposition of ice-rafted debris (associated with calving-front dynamics, ice-shelf basal melt, and transiting icebergs) [142,143].

Although all sites in the study occurred within a polynya, and therefore an area of potentially elevated primary production and related organic matter export to the seabed [74,144], megafaunal density values were not necessarily as high as those recorded for other open-water continental shelf areas (e.g., [42]; and see Table VI of Post et al. [124]). The lower faunal densities observed could be related to the fact that the polynya only occurs intermittently, and the study area is otherwise covered in heavy year-round sea ice. Furthermore, there is a contrasting seasonality of sea-ice cover in the polynya compared to other Southern Ocean areas—sea-ice cover in the polynya being at a maximum during the austral summer (when phytoplankton blooms are typically most productive) as opposed to winter. Such aspects may compromise primary production potential, and thus associated organic matter flux to the Antarctic seabed.

Biological patterns were not only explored by looking at the megafaunal assemblage as a whole, but also through the 12 functional groups represented by the fauna detected (Table 2; Figure 5). Such an approach has advantages in examining the functionality of biodiversity and also potentially helping reduce imagery appraisal times. In the study, specific taxa and functional groups, e.g., anemones and hydroids—both components of the soft sessile predator/scavengers group, showed significant density increases as distance from the ice shelf increased. Some taxa, e.g., echinoids of grazing and deposit-feeding types, showed exceptionally high densities at the Bawden Ice Rise site (LCP1), and low densities (or absences) elsewhere, while others, e.g., climax suspension feeders, showed no discernible trends. It could be predicted that pioneer species would be of higher density in more-recently ice-shelf-uncovered areas and climax species in more established areas (i.e., those that have been uncovered for longer) [48,49]. However, the study found high pioneer densities at LCP4, free of ice-shelf cover during and potentially before the satellite era, and LCP1, the site most consistently ice-shelf-covered and still situated very close to the ice-shelf edge. In addition, pioneer suspension feeders displayed a significant positive density relationship with distance from the ice-shelf edge, particularly when the Jason Peninsula sites were considered exclusively. Climax suspension feeders, e.g., sponges and bryozoans, did not show a trend with distance from the ice shelf, and interestingly, the highest densities were found within an LCP1 sample, challenging the assumption that climax suspension feeders would be more prevalent in areas farther from the ice shelf where primary production may be higher. Additionally, it could also be predicted that grazers would be better represented in seabed spaces featuring more hard substratum (hence potential attached food sources), and deposit-feeders in soft-sedimented areas [1]. While this was not the case for grazers in this study, densities of epifaunal deposit-feeders did increase with increasing substratum softness.

Partitioning faunal density and richness into various taxonomic levels, or by function, permits further insight into how different elements of biodiversity respond to environmental conditions. Finding appropriate surrogacy, or indicators, can also help simplify ecosystem monitoring, accelerate data processing and results-delivery, and thus enhance the timeliness of informing marine management, which is particularly important for rapidly changing environments. Suitable surrogates should reflect the overall biotic pattern of interest while not driving it. In the present study, deposit-feeding crawlers, all of which were holothurians, were found to be the strongest surrogacy candidate for overall megafaunal density (Figure 6). Although preliminary, this finding could be especially advantageous for imagery-based seafloor assessments given that holothurians are relatively easy to detect and identify due to their visual characteristics and mobility, making them also potentially ideal candidates for the development of automated appraisal and monitoring tools (e.g., [145]). Nevertheless, it remains important to continue testing proposed surrogates. For example, gastropods have been used as indicators of latitudinal trends in the reproductive strategy of benthic invertebrates, i.e., Thorson’s rule [146], yet when exploring this rule in echinoderms [147], quite different patterns were uncovered; rather than brooding becoming a more dominant reproductive strategy with increasing latitude, lecithotrophy (non-feeding larvae) became more common. Additionally, in Southern Ocean seabed conservation and management, taxa considered representative of Vulnerable Marine Ecosystems (VMEs) [148,149] can be employed to pinpoint and designate VMEs. While such taxa are invaluable tools, it is not yet fully known whether all are true indicators of VMEs and/or are vulnerable themselves (see [4,56,59]).

Ordination techniques, such as nMDS, can help reveal underlying structural differences in biodiversity at various scales, for example, between islands and seamounts [150], along fjords [132], and across depths [118]. Given that offshore sites were considered more likely to have experienced historic disturbance, hence display differing successional/colonization stages, such sites were expected to be the most compositionally dissimilar (within and between respective sites). The nMDS visualization, however, revealed two particular aspects of note. Firstly, variability, in terms of assemblage composition, was similar within sites irrespective of distance from the ice-shelf edge, and secondly, sites closest to the ice shelf were the most compositionally dissimilar. A potential explanation is that the ice-shelf margin is a similarly (or more) variable continental seabed environment to that of offshore. This variability at the ice front may be due to the differing retreat-advance dynamics of the ice shelf, and the varying degrees (and nature) of sediment loading and stochasticity of release, which will impact the seafloor in terms of disturbance and substratum make-up, hence benthic assemblage structures.

One of the striking biological differences between Jason Peninsula site LCP2 and other sites was that it did not appear to host calcaxonian corals, despite the availability of plentiful hard surfaces, i.e., attachment points. However, the distribution of such biota is also thought to be strongly influenced by water depth, coincident iceberg scouring, and the flow of organic matter [151], so these factors may play an important role around this part of the Larsen C Ice Shelf margin. Furthermore, LCP2 was also the only site observed to host elasipod holothurians, which could indicate a food-poor habitat as the taxon is considered well-adapted to food scarcity [140]. Larsen-marginal habitat differences appear complex, perhaps even enigmatic. Nevertheless, the underlying drivers of assemblage differences are ultimately environmental, with various biota elements responding differently.

A priori considerations that ice-shelf proximity may be a major influencer of megafaunal assemblage composition were not directly supported by the BEST analysis findings of this study. However, such a factor (as aforementioned) could be linked to other environmental variables such as prevailing substratum type. As found in other polar locations and farther afield (e.g., [124,132,152,153,154]), substratum type and depth were also found to be important drivers of faunal composition, although the impact of depth is not always clear in the Antarctic owing to wide bathymetric ranges of many benthic species [136]. Given that areas exhibiting hard substratum are expected to be more heavily colonized and display characteristic taxa [153,154], the substratum results of the present study were somewhat counterintuitive. For instance, the “hardest” site, LCP2, had notably low faunal densities, including very few encrusting lithophiles, and corals appeared unrepresented. It is thus likely that further factors were influential at LCP2, including, for example, potentially organic-poor (oligotrophic) bottom currents coming from beneath the ice shelf, as described by Dayton and Oliver [155] for an area on the west flank of the McMurdo Sound, next to Ross Ice Shelf. Alternatively, the substratum at LCP2 (as per above) was indicative of a more frequently disturbed environment in relation to ice-shelf processes and dynamics; hence faunal assemblage components and densities are reflective.

In the polar seas, sea-ice cover (concentration, timing and duration) is a key influencer of seabed biodiversity dynamics, in part through associated impacts on light penetration, primary production, and flux of particulate organic matter to the seafloor [156,157]. However, in this study, sea-ice cover was not significantly coincident with differences in assemblage structure. This result may be because sea-ice cover (on its own) did not sufficiently differentiate between all of the sites and/or the associated variable did not account for periods of ice-shelf cover. Water physico-chemistry was also found to be important in influencing faunal composition. This variable notably included ocean temperature, considered highly likely to be impacted by climate change, with implications for biotic communities, in particular those of coastal habitats and habitats at continental shelf depths [6]. As an assortment of variables, i.e., depth, substratum type and water physico-chemistry, were the best explanatory set of environmental factors, this supports the view that the Antarctic continental shelf is a complex environment. Exploration of further variables/parameters, in particular those related to biological interactions such as predation and competition, may provide additional, potentially important, ecological insights, particularly in the case of likely alterations to benthic assemblages in response to environmental change. Furthermore, extending studies to other ice-shelf-adjacent polynyas (e.g., those pinpointed in [74]) would help determine if there is a degree of commonality (or not) among polynyas in terms of benthic assemblage structuring and drivers, and, together with the present study, additionally offer a collective ecological baseline for conservation and management purposes.

## 5. Conclusions

This study explored benthic assemblages of four western Weddell Sea continental shelf sites, all within an ice-shelf-adjacent coastal polynya, and two of which have recently experienced ice-shelf retreat. The research revealed distinct faunal patterns in density (but not in richness), and identified a taxonomic and functional group (deposit-feeding crawlers (all holothurians)) that could potentially be a surrogate for the wider megafauna, hence contributing to simplifying, and potentially expediting, future marine environmental monitoring efforts. Distance from the ice shelf was not identified as a key driver of assemblage composition; however, results evidenced that at the ice-shelf front, habitats and assemblages can be very different across space. The study further showed that different environmental variables were potentially important in structuring benthic assemblages at each study site (e.g., water physico-chemistry at LCP1), emphasizing the complexity of Antarctic continental shelf environments, particularly those at the edge of ice shelves.

The current findings provide an indication of how seabed communities may develop along the eastern Antarctic Peninsula as further ice-shelf cover is lost, and narrow biological knowledge gaps of a very rarely accessed and distinctive area within a CCAMLR-proposed MPA.

## Figures and Tables

**Table 1:** Western Weddell Sea continental shelf study sites, LCP1-4, investigated for benthic megafaunal assemblages, including date(s) sampled (seafloor imagery collection), position in decimal degrees latitude (°S) and longitude (°W), and water depth (m) (all means across associated seafloor imagery), and distance from the ice-shelf front at the time of sampling (m).

Site	Date(s) Sampled	Latitude (°S)	Longitude (°W)	Water Depth (m) ^1^	Distance from Ice-Shelf Front (m) ^2^
LCP1—Bawden Ice Rise	21–22 January 2019	−66.6975	−60.2981	397	777
LCP2—Jason Peninsula	20 January 2019	−66.1868	−60.4742	299	2752
LCP3—Jason Peninsula	14 January 2019	−66.0219	−60.3526	400	9024
LCP4—Jason Peninsula	23 January 2019	−66.1544	−60.1594	349	16,459

^1^ Measured during sampling by high-precision acoustic positioning system (Section 2.3). ^2^ Calculated following sampling using Vector Analysis Tools in QGIS v3.16.6.

**Table 2:** Functional group categories (based on [94,95]) and example taxa discerned in Larsen C Ice Shelf Polynya (current study).

Functional Group	Example Taxa
Deposit-feeding crawlers (epifaunal)	Holothurians
Soft infaunal deposit-feeders	None discerned
Hard burrowing deposit-feeders	Irregular echinoids
Flexible strategists	Ophiuroids
Grazers	Regular echinoids
Soft sessile predator/scavengers ^1^	Pennatulaceans, soft corals, anemones (actiniarians), hydroids
Hard sessile predator/scavengers ^1^	Scleractinian corals, calcaxonian whips, hydrocorals
Soft mobile predator/scavengers	Nemerteans, nudibranchs, octopi
Hard mobile predator/scavengers	Fish, gastropods, asteroids
Arthropod predator/scavengers	Pycnogonids, decapod shrimps
Pioneer sessile suspension feeders	Encrusting bryozoans, ascidians, some polychaetes
Climax sessile suspension feeders	Demosponges, hexactinellids, brachiopods, some bryozoans
Sedentary suspension feeders	None discerned
Mobile suspension feeders	Crinoids

^1^ Can also be considered as “active and passive filter feeders” (see [96,97]).

## Data Availability

The data used in this study are contained within the supplementary material or referenced within the text; additionally, the sea ice and ice-shelf front position data can be found at https://doi.org/10.5067/8GQ8LZQVL0VL, accessed on 15 June 2022 and https://doi.org/10.17863/CAM.54490, accessed on 15 June 2022, respectively.

## Supplementary Materials

The following supporting information can be downloaded at: https://www.mdpi.com/article/10.3390/biology11121705/s1, Figure S1. Example imagery and megabenthic communities observed at the Larsen C Polynya study sites, LCP1-4, western Weddell Sea, Antarctica; Figure S2. Morphotaxa richness with substratum type (increasing hardness) for standardized 100 m^2^ samples across Larsen C Polynya (LCP) study sites, LCP1-4; Figure S3. Exploration of functional groups pioneer sessile suspension feeders and hard sessile predator/scavengers as potential surrogates for the overall faunal density trend; Figure S4. Non-metric multidimensional scaling (nMDS) ordination of Larsen C Polynya samples based on Bray–Curtis similarities calculated from fourth-root transformed morphotaxa density data (2D Stress = 0.05); Figure S5. Dendrogram from hierarchical agglomerative clustering (HAC) of Larsen C Polynya samples using group-average linking of Bray–Curtis similarities calculated from functional group densities data. Similarity profiles (SIMPROF) testing applied to determine significance of divisions (at *p* < 0.05); Table S1. Distances (m) between Larsen C Polynya study sites LCP1-4. Calculated using Vector Analysis Tools in QGIS v3.16.6; Table S2. Larsen C Polynya substratum types. Hardness scale; Table S3. Environmental variables considered in the study and correlations ≥ 95% observed; Table S4. Similarity percentages (SIMPER) analysis displaying percentage contributions of benthic morphotaxa responsible for within-site (LCP1-4) similarities. Cut-off where cumulative percentage contribution reaches 50%; Table S5. Similarity percentages (SIMPER) analysis displaying percentage contributions of benthic morphotaxa responsible for dissimilarities between sites (LCP1-4). Cut-off where cumulative percentage contribution reaches 50%.
