# Supplementary material for: Antarctic Seabed Assemblages in an Ice-Shelf-Adjacent Polynya, Western Weddell Sea
Source: Biology (Basel). 2022 Nov 25;11(12):1705. doi: 10.3390/biology11121705 (PMC9774262; doi:10.3390/biology11121705)
Supplement: Supplementary file 1 [file biology-11-01705-s001.zip › biology-2027289-SI.pdf]

Supplementary Material

Antarctic Seabed Assemblages in an Ice-Shelf-Adjacent Polynya, Western Weddell Sea

Frinault et al. 2022. MDPI Biology.

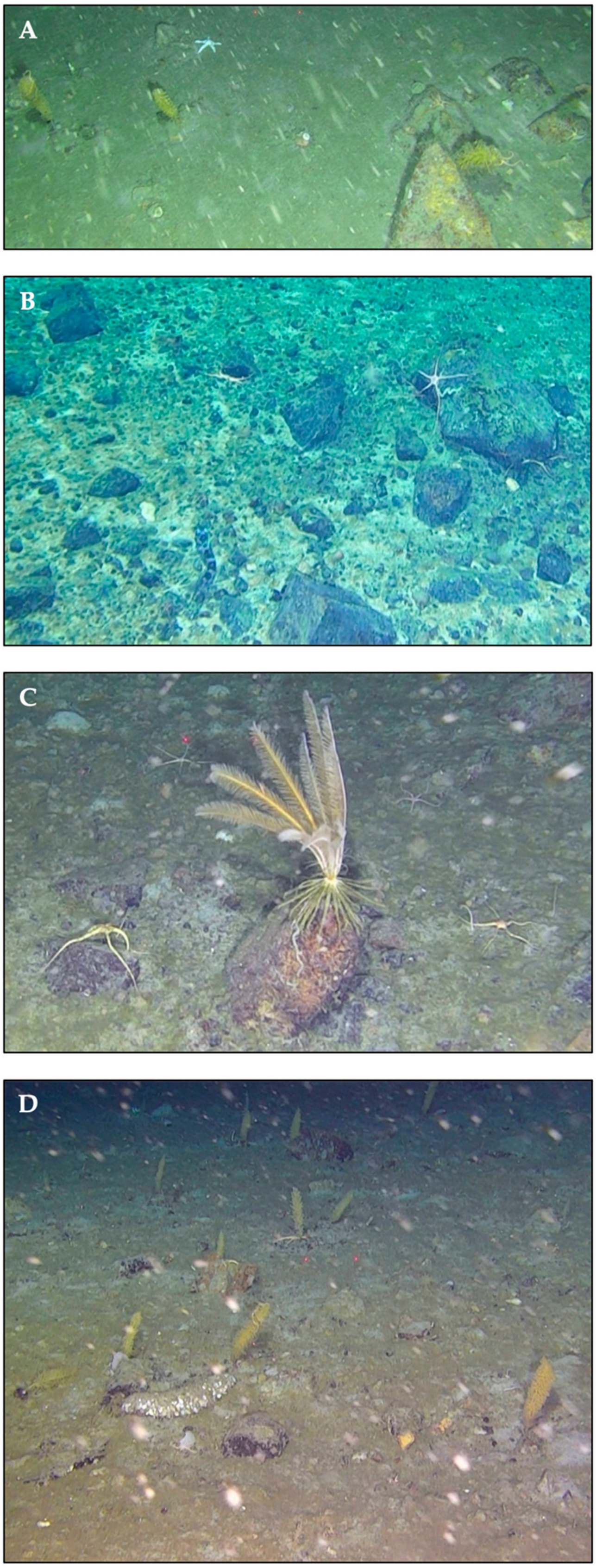

Figure S1. Example imagery and megabenthic communities observed at the Larsen C Polynya (LCP) study sites, LCP1-4, western Weddell Sea, Antarctica: (**A**) LCP1, (**B**) LCP2, (**C**) LCP3, and (**D**) LCP4.

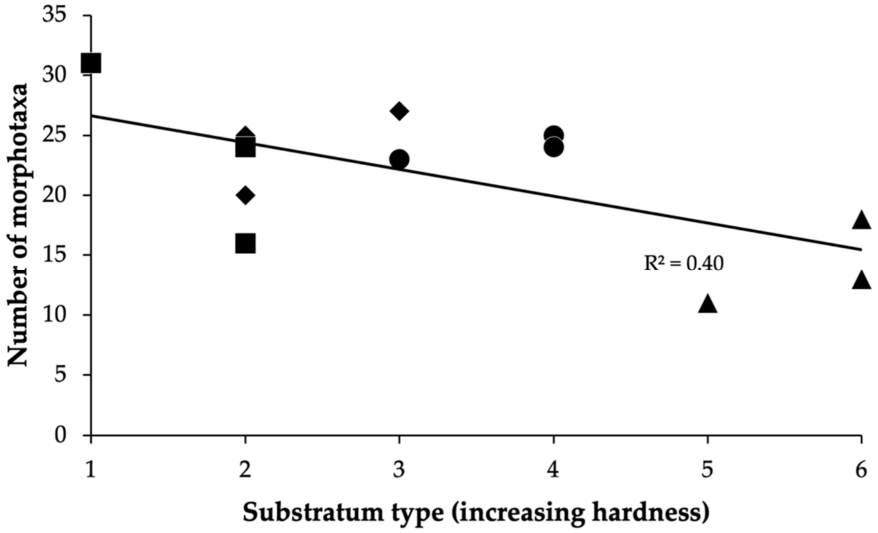

Figure S2. Morphotaxa richness with substratum type (increasing hardness) for standardised 100 m^2 samples across Larsen C Polynya study sites, LCP1-4, and showing a weak (inverse) relationship. Sites are (left to right) LCP4 = squares, LCP1 = diamonds, LCP3 = circles, and LCP2 = triangles.

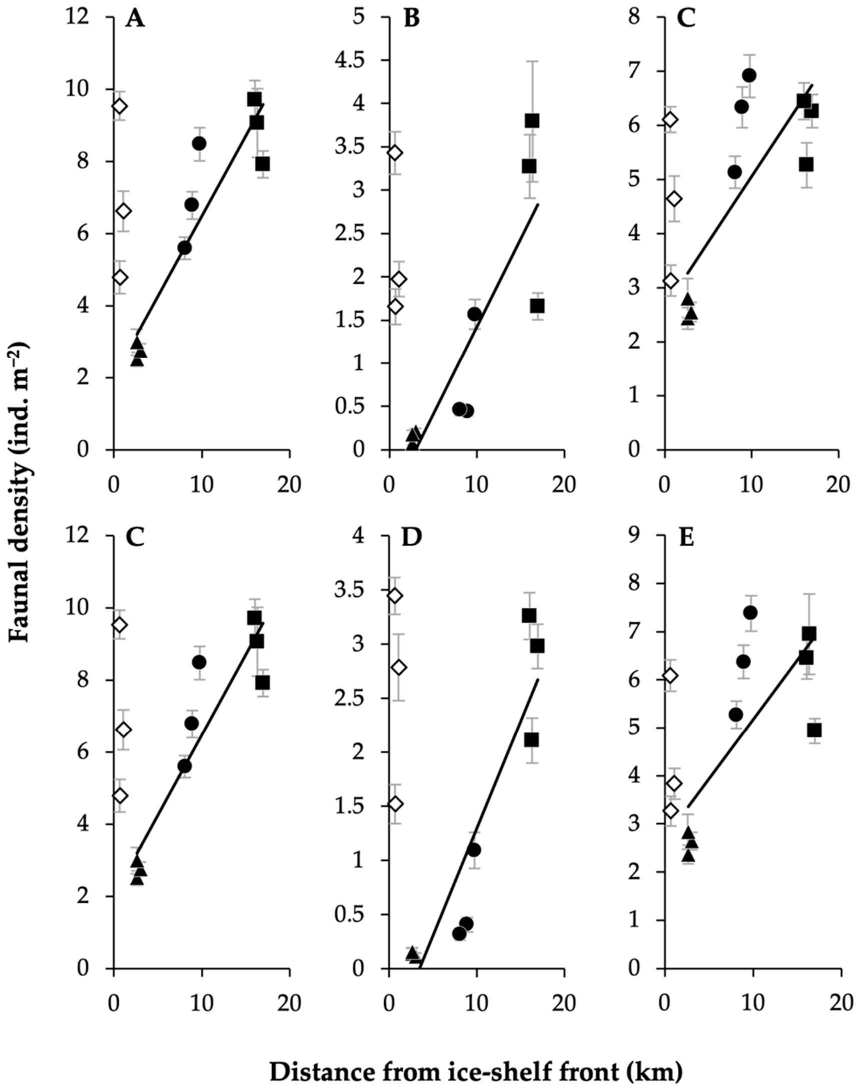

Figure S3. Exploration of functional groups pioneer sessile suspension feeders and hard sessile predator/scavengers as potential surrogates for the overall faunal density trend[a]. Mean densities (ind. m^{-2}) (± SE) with distance from the ice-shelf front (km) of: (A) overall fauna; (B) pioneer sessile suspension feeders; (C) overall fauna excluding pioneer sessile suspension feeders; (D) overall fauna; (E) hard sessile predator/scavengers; and (F) overall fauna excluding hard sessile predator/scavengers. Site LCP1 = diamonds, LCP2 = triangles, LCP3 = circles, and LCP4 = squares.

[a]The overall faunal pattern remains reasonably intact when data of either functional group are removed, however the two functional groups appear to closely match one another in terms of their individual density pattern and their densities influence overall faunal densities. In contrast, the deposit-feeding crawlers group (Figure 6 in the main text) presents densities that do not influence overall faunal densities while reflecting the pattern (hence selected as the strongest surrogacy candidate).

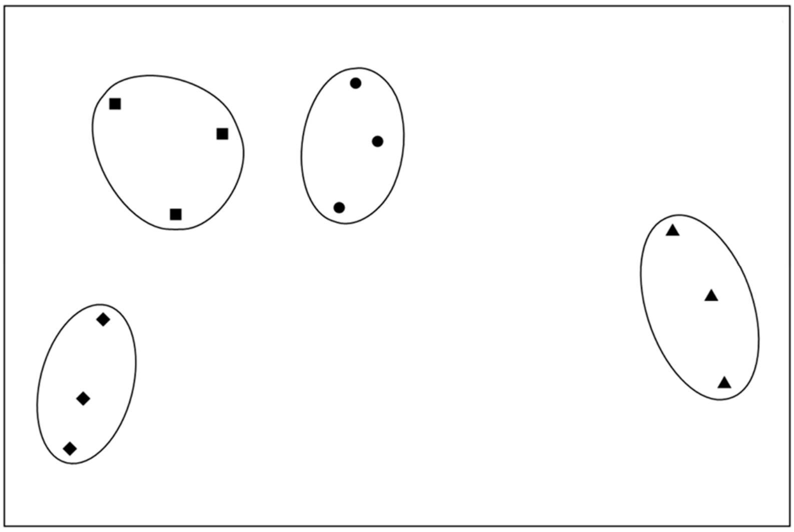

Figure S4. Non-metric multidimensional scaling (nMDS) ordination of Larsen C Polynya samples based on Bray-Curtis similarities calculated from fourth-root transformed morphotaxa density data (2D Stress= 0.05). Symbols for site samples (from left to right) are LCP1 = diamonds, LCP4 = squares, LCP3 = circles, and LCP2 = triangles. Ellipses around samples = significant groupings identified by a SIMPROF test during hierarchical cluster analysis.

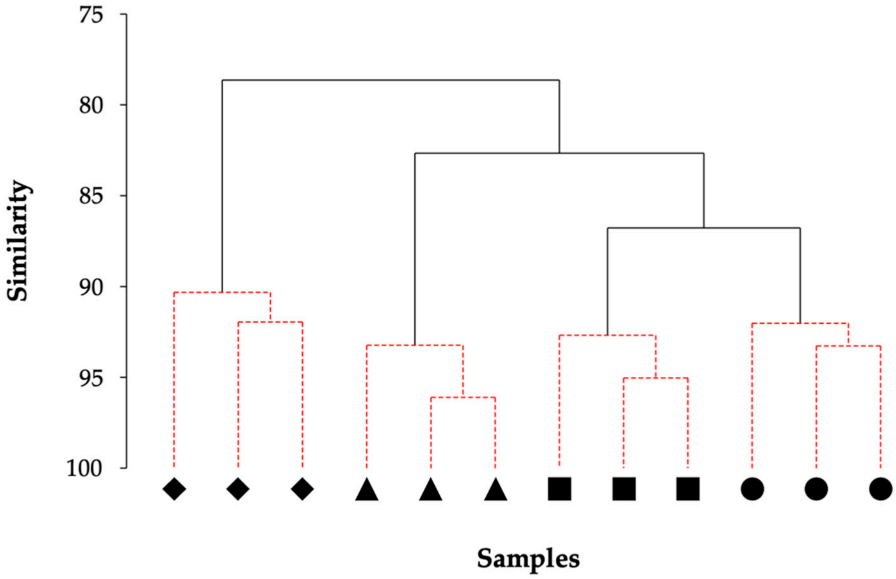

Figure S5. Dendrogram from hierarchical agglomerative clustering of Larsen C Polynya samples using group-average linking of Bray-Curtis similarities calculated from functional group densities data. Similarity profiles (SIMPROF) testing applied to determine significance of divisions (at $p < 0.05$) with black continuous lines indicating significant clusters and red hashed lines indicating non-significant clusters. Site LCP1 = diamonds, LCP2 = triangles, LCP3 = circles, and LCP4 = squares.

Table S1. Distances (m) between Larsen C Polynya study sites LCP1-4. Calculated using Vector Analysis Tools in QGIS v3.16.6.

Site	LCP1	LCP2	LCP3	LCP4
**LCP1**	0	57,490	75,377	60,879
**LCP2**	57,490	0	19,192	14,650
**LCP3**	75,377	19,192	0	17,167
**LCP4**	60,879	14,650	17,167	0

Table S2. Larsen C Polynya substratum types. Hardness scale[a].

Dominant substratum 1	Dominant substratum 2	Hardness scale
Soft sediment	Mixed	**1**
Mixed	Soft sediment	**2**
Mixed	Boulder	**3**
Assorted hard	Mixed	**4**
Assorted hard	Boulder/mixed	**5**
Assorted hard	Boulder	**6**

[a]Soft sediment (predominantly fine-grained sediment, i.e., mud/sand); mixed (patches of both soft and hard sediment); boulder (dominant feature in frame); and assorted hard (sediment of various coarse grain-sizes, i.e., pebbles to boulder).

Table S3. Range of environmental variables[a-e] prepared and examined to discern how sites differ in terms of environmental context. Presented are variables that displayed variation between sites, with variables showing high correlation (or anticorrelation) (r ≥95% [or ≤-95%]) with another identified by ✓. Variables used in statistical analyses are presented (**in bold**) and include variables used as a proxy for respective correlates.

**Data sources**	**GIS**	**ROV**	**Imagery**		**Remote Sensing[b,c]**					**CTD[d,e]**					
**Environmental variables[a]**	**Distance from ice shelf**	**Depth**	**Substratum**	**Phytodetritus**	Mean daily NPP	Mean peak NPP	**Sea ice**	Years ice-shelf-free	Mean ice cover[f]	Silicic acid	**Nitrate**	**Phosphate**	**Salinity**	Oxygen	**Temp**
**Distance from ice shelf**					✓	✓									
**Depth**															
**Substratum**															
**Phytodetritus**															
Mean daily NPP	✓										✓				
Mean peak NPP	✓														
**Sea ice**								✓		✓					
Years as ice-shelf-free							✓								
Mean ice cover[f]															
Silicic acid							✓								
**Nitrate**					✓										
**Phosphate**															
**Salinity**															
Oxygen															✓
**Temp**														✓	

[a]Distance from ice shelf = distance of sample from the ice-shelf front at time of sampling (m); Depth = mean depth of sample (m); Substratum = substratum type hardness of sample by dominating substratum types (as per Table S2); Phytodetritus = degree of phytodetritus cover (1 to 5; 1 = lowest, 5 = highest); Mean daily NPP = mean daily net primary production over the period 1997-2018 (mg C m^{-2} d^{-1}); Mean peak NPP = mean net peak primary production over the period 1997-2018 (mg C m^{-2} d^{-1}); Sea ice = mean monthly sea-ice concentration (SIC) over the period 1997-2018 (%); Number of years ice-shelf-free = number of years not covered by ice shelf for the period 1963-2018; Mean ice cover = composite mean monthly ice cover (incorporating both ice-shelf cover and sea-ice concentration data) for the period 1997-2018 (%) (not used in the main study); Silicic acid = Log

[Silicic acid] (μM); Nitrate = Log [Nitrate] (μM); Phosphate = Log [Phosphate] (μM); Salinity = Salinity (PSU); Oxygen = Log [Oxygen] (μM); Temp = bottom water temperature (°C).

Correlations: Distance from ice shelf with mean daily NPP and mean peak NPP; nitrate with mean daily NPP; sea ice with silicic acid and years ice-shelf-free; and temp with oxygen (represented in the study as "water physico-chemistry", and indicating LCP1 to be situated in warmer and less oxygenated waters than LCP2-4).

[b]Mean daily NPP and mean peak NPP were determined from data generated as per procedures described in Arrigo, K.R.; van Dijken, G.L.; Bushinsky, S. Primary production in the Southern Ocean, 1997–2006. *J. Geophys. Res.* **2008**, 113, doi:10.1029/2007jc004551 and Arrigo, K.R.; van Dijken, G.L.; Strong, A.L. Environmental controls of marine productivity hot spots around Antarctica. *J. Geophys. Res. Oceans* **2015**, 120, 5545-5565, doi:10.1002/2015jc010888.

[c]Sea-ice concentration and number of years ice-shelf-free (and mean ice cover) were based on data obtained as per methods described in Christie, F.D.W.; Benham, T.J.; Batchelor, C.L.; Rack, W.; Montelli, A.; Dowdeswell, J.A. Antarctic ice-shelf advance driven by anomalous atmospheric and sea-ice circulation. *Nat. Geosci.* **2022**, 15, 356-362, doi:10.1038/s41561-022-00938-x.

[d]Nitrate, silicic acid, and phosphate concentrations were acquired as per methodologies described in Flynn, R.F.; Bornman, T.G.; Burger, J.M.; Smith, S.; Spence, K.A.M.; Fawcett, S.E. Summertime productivity and carbon export potential in the Weddell Sea, with a focus on the waters adjacent to Larsen C Ice Shelf. *Biogeosciences* **2021**, 18, 6031-6059, doi:10.5194/bg-18-6031-2021.

[e]Salinity and temperature measurements, and oxygen concentrations were obtained as per methods described in Hutchinson, K.; Deshayes, J.; Sallee, J.B.; Dowdeswell, J.A.; Lavergne, C.; Ansorge, I.; Luyt, H.; Henry, T.; Fawcett, S.E. Water Mass Characteristics and Distribution Adjacent to Larsen C Ice Shelf, Antarctica. *J. Geophys. Res. Oceans* **2020**, 125, doi:10.1029/2019jc015855.

[f]Mean ice cover: To take into consideration both types of ice cover, i.e., sea-ice and ice-shelf cover, a composite variable: "mean ice cover" (%), was computed using SIC (%) and historical ice-shelf cover data. Mean ice cover was calculated (for each site) for the 1997-2018 period and wherein ice-shelf cover corresponded to 100% ice cover. Although not used in the main paper, when mean ice cover was trialed in BEST analysis, the measure was found to best correlate (along with substratum) with benthic assemblage composition differences.

Table S4. Similarity percentages (SIMPER) analysis displaying percentage contributions of benthic morphotaxa responsible for within-site (LCP1-4) similarities. Cut-off where cumulative percentage contribution reaches 50%. Overall average similarities (%): LCP1 samples = 71.57; LCP2 samples = 69.82; LCP3 samples = 70.94; LCP4 samples = 74.3.

**Benthic morphotaxa**	**Av. dens. (ind. m^{-2})**	**Av. sim. (%)**	**Sim./SD**	**Contrib. (%)**	**Cum. contrib. (%)**
**LCP1**					
Bottlebrush/bushy corals - (e.g., *Thouarella*)	1.22	8.57	12.36	11.98	11.98
Encrusting lithophilic organisms	1.16	8.19	14.63	11.44	23.42
Ophiuroidea	0.96	6.77	11.59	9.47	32.89
Echinoid 1 - *Sterechinus antarcticus*	0.74	5.25	14.08	7.34	40.23
Asteroidea					
Holothurian 1 - *Pseudostichopus mollis*	0.62	4.42	13.17	6.18	53.06
**LCP2**					
Ophiuroidea	1.16	15.17	19.34	21.72	21.72
Encrusting lithophilic organisms	0.61	7.55	7.35	10.81	32.53
Scleractinian cup corals (e.g., Flabellidae)	0.58	7.38	35.84	10.57	43.1
Asteroidea					
**LCP3**					
Ophiuroidea	1.44	11.67	117.16	16.45	16.45
Encrusting lithophilic organisms	0.88	6.55	33.04	9.23	25.68
Holothurian 1 - *Pseudostichopus mollis*	0.72	5.8	102.97	8.18	33.86
Bottlebrush/bushy corals - (e.g., *Thouarella*)	0.71	5.22	31.09	7.35	41.21
Asteroidea	0.64	4.96	17.34	7	48.21
Scleractinian cup corals (e.g., Flabellidae)	0.64	4.95	21.11	6.97	55.18
**LCP4**					
Bottlebrush/bushy corals - (e.g., *Thouarella*)	1.26	8.63	102.99	11.62	11.62
Ophiuroidea	1.18	8.34	26.47	11.22	22.84
Encrusting lithophilic organisms	1.21	8.24	9.8	11.1	33.93
Holothurian 1 - *Pseudostichopus mollis*	0.77	5.09	7.59	6.85	40.78
Asteroidea					
Ascidian 1 - *Pyura* spp.					

Av. dens. = average density, Av. sim. = average similarity, Sim./SD = similarity/standard deviation, Contrib. = contribution, Cum. contrib. = cumulative contribution.

Table S5. Similarity percentages (SIMPER) analysis displaying percentage contributions of benthic morphotaxa responsible for dissimilarities between sites (LCP1-4). Cut-off where cumulative percentage contribution reaches 50%. Overall average dissimilarities (%): LCP1 and LCP2 = 61.20; LCP1 and LCP3 = 47.36; LCP1 and LCP4 = 41.56; LCP2 and LCP3 = 49.23; LCP2 and LCP4 = 56.90; LCP3 and LCP4 = 38.72.

Benthic morphotaxa	Av. dens. (ind. m^{-2})	Av. dens. (ind. m^{-2})	Av. diss. (%)	Diss./SD	Contrib. (%)	Cum. contrib. (%)
**Sites closest to ice-shelf front**	**LCP1**	**LCP2**				
Bottlebrush/bushy corals (e.g., *Thouarella*)	1.22	0	5.88	14.53	9.61	9.61
Encrusting lithophilic organisms	1.16	0.61	2.63	7.49	4.3	13.91
Gymnolaemate bryozoans	0.53	0	2.55	15.63	4.16	18.07
Echinoid 1 - *Sterechinus antarcticus*	0.74	0.28	2.2	11.11	3.59	21.66
Ascidian 1 - *Pyura* spp.	0.43	0	2.13	2.9	3.48	25.14
Echinoid 3 - Spatangoida	0.43	0	2.1	5.07	3.43	28.57
Holothurian 2 - *Peniagone vignoni*	0	0.42	2.03	6.71	3.31	31.88
Echinoid 2 - Ctenocidaridae	0.41	0	1.97	21.44	3.23	35.11
Holothurian 1 - *Pseudostichopus mollis*	0.62	0.23	1.86	1.96	3.04	38.15
Demosponge – *Phakellia*	0.38	0	1.79	7.85	2.92	41.07
Perciformes - Bathydraconidae	0.33	0	1.58	7.31	2.58	43.65
Demosponge 2	0.33	0	1.52	1.23	2.48	46.12
Whip corals	0.29	0	1.49	1.33	2.44	48.56
Decapod shrimps	0.31	0	1.48	20.73	2.42	50.98
**Sites at ~400 m depth**	**LCP1**	**LCP3**				
Echinoid 1 - *Sterechinus antarcticus*	0.74	0.21	2.08	3.24	4.4	4.4
Bottlebrush/bushy corals (e.g., *Thouarella*)	1.22	0.71	2.01	3.25	4.24	8.64
Ophiuroidea	0.96	1.44	1.91	4.36	4.03	12.66
Echinoid 3 - Spatangoida	0.43	0	1.72	5.36	3.63	16.29
Echinoid 2 - Ctenocidaridae	0.41	0	1.62	17.49	3.42	19.72
Demosponge – *Phakellia*	0.38	0	1.47	6.95	3.1	22.82
Stalked crinoids (e.g., *Dumetocrinus antarcticus*)	0	0.33	1.33	5.95	2.8	25.62
Eucarid malacostracans	0	0.33	1.32	8.56	2.78	28.4
Perciformes - *Bathydraconidae*	0.33	0	1.29	7.62	2.73	31.14
Demosponge 2	0.33	0	1.25	1.22	2.65	33.78
Demosponge 1	0.31	0	1.21	21.14	2.56	36.35
Sea pen 2 - *Umbellula* spp.	0	0.3	1.2	7.78	2.54	38.89
Stenolaemate bryozoans	0.29	0	1.12	1.28	2.37	41.26
Encrusting lithophilic organisms	1.16	0.88	1.09	1.74	2.31	43.57
Unstalked crinoids (e.g., *Promachocrinus kerguelensis*)	0.2	0.45	1.01	1.46	2.13	45.7
Hexactinellid 1 - *Chonelasma*	0	0.25	1	1.28	2.11	47.81
Hexactinellida	0.24	0	0.94	1.32	2	49.8
Ascidian 1 - *Pyura* spp.	0.43	0.22	0.94	1.33	1.99	51.79
	**LCP1**	**LCP4**				
Echinoid 1 - *Sterechinus antarcticus*	0.74	0	2.71	17.89	6.52	6.52
Eucarid malacostracans	0	0.46	1.69	6.58	4.08	10.6
Hydroid 1	0.11	0.55	1.65	2.32	3.98	14.57
Echinoid 3 - Spatangoida	0.43	0.09	1.23	2.23	2.95	17.53
Perciformes - Bathydraconidae	0.33	0	1.2	7.6	2.88	20.41
Echinoid 2 - Ctenocidaridae	0.41	0.09	1.14	2.29	2.74	23.14
Nudibranchia	0	0.31	1.13	8.57	2.73	25.87

Demosponge 1	0.31	0	1.12	18.91	2.7	28.57
Demosponge 2	0.33	0.13	1.06	1.22	2.56	31.13
Stenolaemate bryozoans	0.29	0	1.04	1.27	2.51	33.64
Demosponge – *Phakellia*	0.38	0.1	1.01	1.7	2.44	36.08
Gymnolaemate bryozoans	0.53	0.26	0.97	1.34	2.33	38.41
Soft coral 1 - Nephtheidae	0.1	0.36	0.93	1.73	2.25	40.65
Sabelleria	0.09	0.32	0.85	1.55	2.05	42.7
Sea pen 1	0	0.23	0.84	1.33	2.02	44.73
Ophiuroidea	0.96	1.18	0.83	2.41	2.01	46.73
Unstalked crinoids (e.g., *Promachocrinus kerguelensis*)	0.2	0.42	0.81	1.32	1.95	48.68
Holothurian 3 - *Bathyplotes gourdoni*	0.23	0	0.8	1.31	1.93	50.61
	**LCP2**	**LCP3**				
Bottlebrush/bushy corals (e.g., *Thouarella*)	0	0.71	3.63	7.57	7.38	7.38
Decapod shrimps	0	0.51	2.6	19.4	5.28	12.66
Holothurian 1 - *Pseudostichopus mollis*	0.23	0.72	2.5	2.57	5.09	17.75
Holothurian 2 - *Peniagone vignoni*	0.42	0	2.14	7.74	4.35	22.09
Gymnolaemate bryozoans	0	0.3	1.55	8.46	3.15	25.24
Whip corals	0	0.29	1.46	21.9	2.96	28.2
Ophiuroidea	1.16	1.44	1.4	8.15	2.84	31.05
Encrusting lithophilic organisms	0.61	0.88	1.39	1.92	2.82	33.87
Holothurian 3 - *Bathyplotes gourdoni*	0	0.26	1.3	1.33	2.65	36.52
Hexactinellid 1 - *Chonelasma*	0	0.25	1.29	1.29	2.62	39.14
Demospongiae	0	0.25	1.26	1.33	2.56	41.7
Unstalked crinoids (e.g., *Promachocrinus kerguelensis*)	0.22	0.45	1.18	1.28	2.4	44.1
Ascidian 1 - *Pyura* spp.	0	0.22	1.14	1.33	2.31	46.41
Hydrozoan 1 - Rhodaliidae	0.22	0	1.09	1.28	2.22	48.63
Brisingids	0.21	0	1.08	1.33	2.2	50.83
**Sites at <350 m depth**	**LCP2**	**LCP4**				
Bottlebrush/bushy corals (e.g., *Thouarella*)	0	1.26	5.81	45.29	10.22	10.22
Ascidian 1 - *Pyura* spp.	0	0.6	2.8	6.84	4.93	15.15
Encrusting lithophilic organisms	0.61	1.21	2.8	5.77	4.93	20.07
Hydroid 1	0	0.55	2.55	9.01	4.48	24.56
Holothurian 1 - *Pseudostichopus mollis*	0.23	0.77	2.5	2.56	4.38	28.94
Holothurian 2 - *Peniagone vignoni*	0.42	0	1.94	7.46	3.41	32.35
Whip corals	0	0.39	1.81	4.7	3.18	35.52
Soft coral 1 - Nephtheidae	0	0.36	1.64	8.27	2.88	38.4
Hexactinellida	0	0.33	1.55	17.21	2.72	41.12
Sabelleria	0	0.32	1.47	9.45	2.58	43.7
Echinoid 1 - *Sterechinus antarcticus*	0.28	0	1.31	9.46	2.3	46
Gymnolaemate bryozoans	0	0.26	1.23	1.3	2.15	48.16
Decapod shrimps	0	0.25	1.12	1.32	1.97	50.13
**Sites furthest from ice-shelf front**	**LCP3**	**LCP4**				
Bottlebrush/bushy corals (e.g., *Thouarella*)	0.71	1.26	2.09	4.18	5.39	5.39
Hydroid 1	0.12	0.55	1.66	2.24	4.27	9.67
Ascidian 1 - *Pyura* spp.	0.22	0.6	1.47	2.21	3.79	13.46
Hexactinellida	0	0.33	1.28	17.82	3.3	16.76
Stalked crinoids (e.g., *Dumetocrinus antarcticus*)	0.33	0	1.28	6.3	3.3	20.07

Encrusting lithophilic organisms	0.88	1.21	1.27	2	3.29	23.36
Sabelleria	0	0.32	1.21	9.99	3.13	26.48
Decapod shrimps	0.51	0.25	1.02	1.28	2.62	29.11
Holothurian 3 - *Bathyplotes gourdoni*	0.26	0	0.98	1.33	2.54	31.65
Ophiuroidea	1.44	1.18	0.97	7.28	2.5	34.15
Hexactinellid 1 - *Chonelasma*	0.25	0	0.96	1.29	2.49	36.64
Scleractinian cup corals (e.g., *Flabellidae*)	0.64	0.4	0.93	3.06	2.41	39.05
Sea pen 1	0	0.23	0.88	1.33	2.27	41.32
Anemone 1	0.15	0.21	0.83	1.35	2.14	43.45
Echinoid 1 - *Sterechinus antarcticus*	0.21	0	0.82	1.31	2.11	45.56
Nudibranchia	0.09	0.31	0.81	1.52	2.1	47.66
Actiniaria/Corallimorpharia/Ceriantharia	0.22	0.4	0.79	1.23	2.04	49.7
Ascidian 2	0.18	0	0.71	1.33	1.85	51.55

Av. dens. = average density, Av. diss. = average dissimilarity, Diss./SD = dissimilarity/standard deviation, Contrib. = contribution, Cum. contrib. = cumulative contribution.
